# Transcriptomics and co-expression network analysis revealing candidate genes for the laccase activity of *Trametes gibbosa*

**DOI:** 10.1186/s12866-022-02727-3

**Published:** 2023-01-26

**Authors:** Jie Chen, Yi Ye, Yujie Chi, Xin Hao, Qingquan Zhao

**Affiliations:** 1 Northeast Forestry University, Harbin, China; 2 Wageningen University & Research, Wageningen, Netherlands

**Keywords:** White-rot fungi, Laccase, WGCNA, Mitochondria

## Abstract

**Background:**

*Trametes gibbosa*, which is a white-rot fungus of the Polyporaceae family found in the cold temperate zone, causes spongy white rot on wood. Laccase can oxidize benzene homologs and is one of the important oxidases for white rot fungi to degrade wood. However, the pathway of laccase synthesis in white rot fungi is unknown.

**Results:**

The peak value of laccase activity reached 135.75 U/min/L on the 9th day. For laccase activity and RNA-seq data, gene expression was segmented into 24 modules. Turquoise and blue modules had greater associations with laccase activity (positively 0.94 and negatively -0.86, respectively). For biology function, these genes were concentrated on the cell cycle, citrate cycle, nicotinate, and nicotinamide metabolism, succinate dehydrogenase activity, flavin adenine dinucleotide binding, and oxidoreductase activity which are highly related to the laccase synthetic pathway. Among them, gene_8826 (MW199767), gene_7458 (MW199766), gene_61 (MW199765), gene_1741 (MH257605), and gene_11087 (MK805159) were identified as central genes.

**Conclusion:**

Laccase activity steadily increased in wood degradation. Laccase oxidation consumes oxygen to produce hydrogen ions and water during the degradation of wood. Some of the hydrogen ions produced can be combined by Flavin adenine dinucleotide (FAD) to form reduced Flavin dinucleotide (FADH2), which can be transmitted. Also, the fungus was starved of oxygen throughout fermentation, and the NADH and FADH2 are unable to transfer hydrogen under hypoxia, resulting in the inability of NAD and FAD to regenerate and inhibit the tricarboxylic acid cycle of cells. These key hub genes related to laccase activity play important roles in the molecular mechanisms of laccase synthesis for exploring industrial excellent strains.

**Supplementary Information:**

The online version contains supplementary material available at 10.1186/s12866-022-02727-3.

## Introduction

Microbial degradation of wood can effectively avoid environmental pollution caused by chemicals. White-rot fungi have been extensively studied as one of the most effective microorganisms for lignin degradation. It can decompose lignocellulose by secreting lignin-decomposing enzymes, such as manganese peroxidase, lignin peroxidase, and multifunctional peroxidase and laccase [1, 2]. *Trametes gibbosa* is a white-rot fungus widely distributed in the cold temperate zone. *T. gibbosa* is characterized by a fast growth rate and strong ability to decompose wood and can decompose lignin by an enzymatic system in which the lignocellulolytic enzyme system is comprised of laccase and Mn-dependent peroxidase, as well as a series of cellulase and cellobiose dehydrogenase [3, 4]. It also has some auxiliary enzymes, including glyoxal oxidase, aryl alcohol oxidases, cellobiose dehydrogenase, and glucose oxidase in the genome [5–7]. Many redox enzymes of the lignin decomposition enzyme system have not been studied.

Laccase is a multicopper oxidoreductase containing four copper ions and is present in plants, fungi, insects, and bacteria [8–11]. In fungi, laccase has many physiological functions, including defense [12], melanin synthesis, and lignin degradation [13]. Recent studies have indicated that laccase has a high application value and can be used in paper bleaching, as a biological fertilizer, and in the degradation of refractory pollutants [14–16]. In the redox cycle, it uses O2 as the final electron acceptor, and the substrate is oxidized to produce unstable phenoxy radicals and/or quinone intermediates, which then initiate spontaneous polymerization into macromolecular products [17]. With the development of modern molecular biology, the researcher began to focus on the study of genes and proteins. Laccase genes were cloned, such as from *Phanerochaete chrysosporium* [18], *Fomes diagnosis* [19], *Pycnoporus cinnabarinus* [20], and *Trametes* sp. [21], to produce more efficiently than endogenous proteins. Also, there are many types of laccase proteins, *Lcc_1* to *Lcc_8* laccase proteins showed regions of high amino acid homology interrupted. All of them have ten conserved histidine and a conserved cysteine in the active center [22]. Cloning of laccase genes alone is insufficient, and comprehensive metabolic systems should be explored to understand the synthetic pathway of laccase in the cells and this facilitates their biotechnological application.

Weighted gene co-expression network (WGCNA) is used to describe the correlation and visualization of data points [23]. Related pathways and genes that play an important role in transcriptional regulation can easily predict regulatory relationships of unknown genes based on the known genes in a network. The method is widely used in humans, animals, and plants[24–26].

In wild, *T. gibbosa* secretes manganese peroxidase and laccase to participate in wood decay and litter degradation [27, 28]. In this study, the RNA-seq data analysis of *T. gibbosa* (CB-1) with wood chips (*Populus simonii* × *P.nigra*) was performed. Mycelia was cultured without chips as the CK group. Then, these were collected in 3, 5, 7, and 11 days with chips as the treatment groups. The effect of wood chips on the secondary metabolism of *T. gibbosa* during growth is studied. Exploring the candidate genes and pathways related to laccase activity may be beneficial to enhancing the laccase activity, and provide a theoretical basis for increasing the degradation rate of wood by *T. gibbose*.

## Materials and methods

### Sample mycelium

The *T. gibbosa* strain CB-1 was preserved in the Plant Pathology Laboratory of Northeast Forestry University. Each conical flask with LNAS medium (70 mL of low nitrogen asparagine succinic acid solution and 5 mL of 15% glucose) contained 5 pieces of the fungus plates (diameter 5 mm). They were cultured statically, at 27 ℃. After 10 days, mycelium was collected, as CK. Then, 2 g wood chips (*Populus simonii* × *P. nigra*) (about 5 cm × 3 cm × 0.5 cm) were added to the conical flasks, static culture. After 3, 5, 7, and 11 days, it was extracted and assigned to the MX1, MX2, MX3, and MX4 groups, respectively. Mycelium was scraped from wood chips using a sterile scalpel and rinsed with ddH_2_O. All samples were stored at -80 °C until RNA was extracted. All groups included 3 biological replicate samples, and each biological replicate was composed of 5 flasks.

The laccase activity was determined by an ultraviolet spectrophotometer (Gene Quant TM 1300, Biochrome) at 420 nm to detect the oxidation rate of ABTS (2,2'-diazo (3-ethylbenzothiazoline-6-sulfonic acid). The reaction system (1 mL) contained 850 μL of 50 mmol·L^−1^ sodium malonate buffer, 50 μL of 20 mmol L^−1^ ABTS solution, and 100 μL of the diluted enzyme solution. An increase in absorbance at 420 nm was determined after 3 min. The amount of enzyme required to oxidize 1 nmol of ABTS per minute per liter of liquid was defined as a unit of laccase activity (nmol/min/L) [29].

### RNA sequencing

Mycelium (15-samples of 0.1 g) was ground to break the cell wall, and Trizol reagent (Invitrogen, USA) was used to extract the total RNA. The quality of the RNA was determined by OD_260/230_ and OD_260/280_ ratios by ultraviolet spectrophotometer (Eppendorf AG) and by agarose gel electrophoresis. The RNA concentration was measured using a NanoDrop 2000 (Thermo), and the RNA Nano 6000 detection kit and the Agilent Bioanalyzer 2100 system (Agilent Technologies, California, USA) were used to assess the integrity of RNA.

Use NEBNext Ultra ™ RNA library prep kit for Illumina (NEB#E7530, USA) to create a sequencing library (1 μg per RNA) and add the index code to the appropriate sample sequence. First-strand cDNA was synthesized using random hexamer primers and M-MuLV reverse transcriptase. Second strand cDNA synthesis was subsequently performed using DNA polymerase I and RNase H. Remaining overhangs were converted into the blunt ends using exonuclease/polymerase. After adenylation of the 3’ ends of the DNA fragments, the NEBNext adaptor with a hairpin loop structure was ligated to prepare for hybridization. The library fragments were purified using an AMPure XP system (Beckman Coulter, Beverly, USA) to select cDNA fragments preferentially 240 bp in length. Then incubate 3 μL USER (NEB, USA) and the cDNA connected to the selected adaptor for 15 min at 37℃ and 5 min at 95℃, and then perform Polymerase Chain Reaction (PCR) [30]. PCR was performed using Phusion high-fidelity DNA polymerase, universal PCR primers, and Index (X) primers. Finally, there were purified (AMPure XP system), and library quality was assessed using an Agilent Bioanalyzer 2100 system. After the cluster is created, the library can be ranked on the Illumina platform to generate suitable examples. Cleaned data were obtained by removing the reads containing adapter, reads containing ploy-N and low-quality reads from the raw data. Q20, Q30, GC-content, and sequence duplication levels of the clean data were calculated. TopHat2 (version 5.0.3.12) was used to map the samples to the reference genome (http://genome.jgi.doe.gov/Tragib1/Tragib1.home.html) [31, 32]. Cufflinks software was used to quantify the gene expression levels based on fragments per kilobase of transcript per million mapped reads (FPKM) by comparison with the reference genome [33]. The principal component analysis (PCA) was conducted as transcriptome samples clustered by the ggplot2 package in R.

### Identification of differentially expressed genes (DEGs)

The annotation of genes includes NCBI nonredundant protein (Nr) database (http://www.ncbi.nlm.nih.gov), Kyoto Encyclopedia of Genes and Genomes (KEGG) pathway annotation (https://www.kegg.jp), Clusters of orthologous groups of proteins (COG) functional annotation (http://www.ncbi.nlm.nih.gov/COG), Swiss-Prot protein database (http:// www.expasy.ch/sprot), and Gene Ontology (GO) annotation (http: //geneontology.org/). The edgeR package was used to identify DEGs between the groups based on the absolute value of fold change (FC) ≥ 2 and a false discovery rate (FDR) < 0.05. The enrichment analysis according to the hypergeometric distribution algorithm with the ClusterProfiler package (https://bioconductor.org/).

### Validation of gene expression by Real-Time Quantitative Reverse Transcription PCR (qRT-PCR)

Ten genes were selected for qRT-PCR analysis. Primer 6.0 software was used to design the primers, which were provided by Sangon Biotech (Shanghai, China) (Table S1). The GPD gene was used as the housekeeping gene [34]. Four DEGs and six of the hub genes were selected for qRT-PCR analysis to verify the effectiveness of high-throughput transcriptome sequencing. An Agilent Technologies Mx3000P instrument was used for real-time PCR detection, and qPCR was performed according to the protocol provided by a Takara one-step RT-PCR kit (Baori Medical Technology Co., Ltd., Beijing, China); the gene expression results were processed using the threshold cycle (2^−ΔΔCt^) method. To compare the RNA-Seq and qPCR results, a linear correlation was calculated using Log_2_FC.

### Construction of the weighted gene co-expression network analysis (WGCNA)

**Fig. 1:**
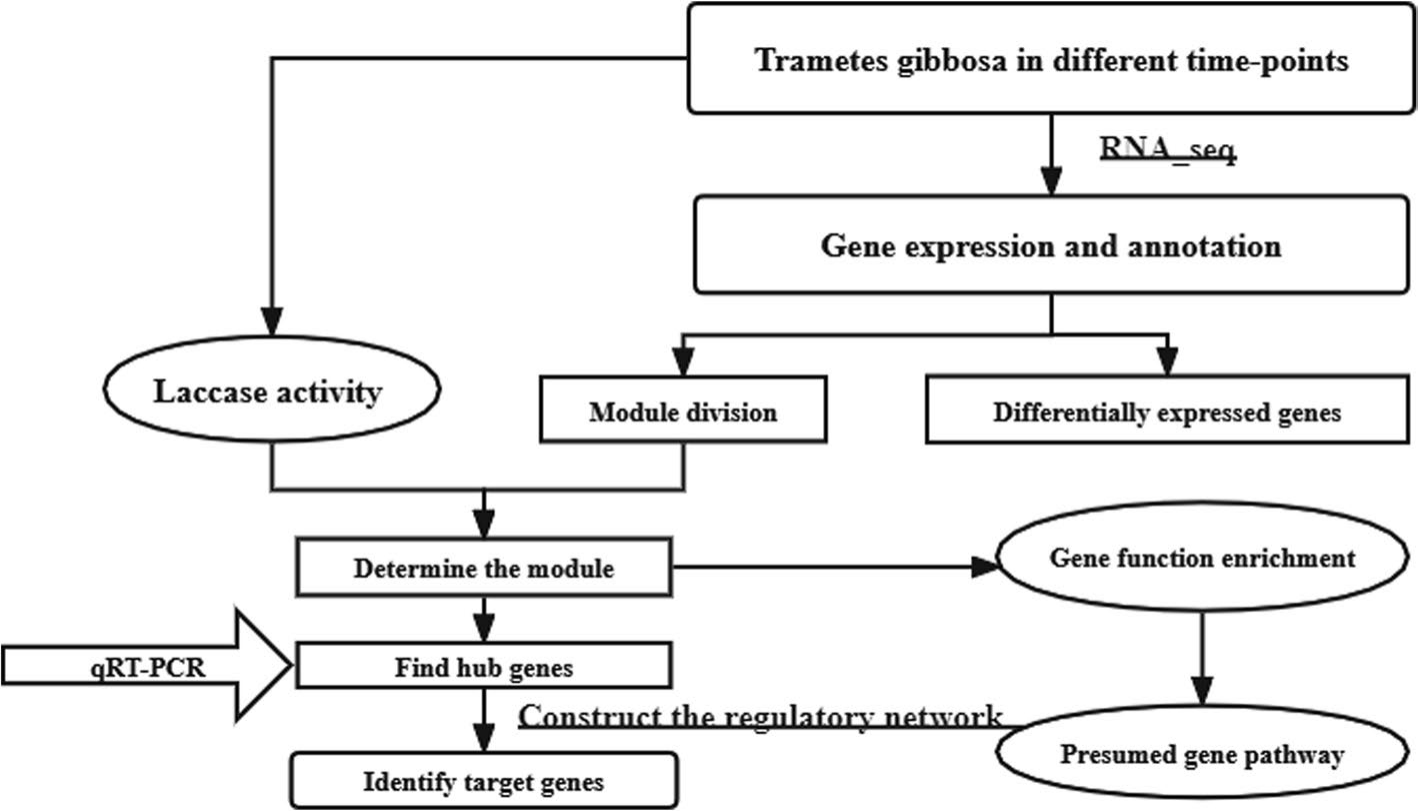
The study design and methodology workflow

A total of 15 samples (5-time points in 3 replicates) were used. Co-expression networks were constructed using the WGCNA (v1.69) package in R [23]. The normalized (screened for missing values) gene expression matrix was imported into WGCNA to construct the co-expression modules. The gene expression matrix was searched for a suitable soft threshold to build gene networks using a scale-free topology model [35]. A threshold filter finally selected β = 22. The adjacency matrix was transformed into a topological overlap matrix (TOM) to evaluate the correlation between gene expression [35], and the dissimilar topological matrix (dissTOM = 1-TOM) was used to carry out the matrix clustering and module partitioning by the dynamic sharing algorithm. The minimum number of elements in a module was 30 (module Size = 30), and the threshold for the merging of a similar module was 0.2 (CutHeight = 0.2). Module eigengenes (MEs) were used to calculate the correlation coefficients to identify the biologically significant modules. The Pearson correlation coefficient of the corPvalueStudent () function was used to calculate the correlations between the laccase matrix and the module feature gene matrix to obtain the p-values. Lower p-values correspond to higher significance of correlations between two matrices. Laccase-related specific modules were identified based on |r|> 0.60 and *p*-value < 0.001 as specific modules for subsequent analysis. The gene expression at different time points was transformed into the same order of magnitude and measured by the calculated Z-Score value to see the changing trend of gene expression using python. The networks were visualized using Cytoscape_3.7.1 (Fig. 1).

### Identification of significant hub genes

Hub genes are generally characterized by high gene significance (GS), high module membership (MM), and high intramodular connectivity (K.in). A hub gene in a module is highly connected with other genes; thus, a hub gene has high functional significance and is often located in the center of a module network [36]. The biological functions of each module were analyzed by enrichment analysis of the GO and KEGG pathways. The protein sequences of each module were submitted to Plant TFDB (http://planttfdb.cbi.pku.edu.cn/prediction.php) for analysis and forecasting to obtain transcription factor predictions [37].

## Results

### Laccase activity

**Fig. 2:**
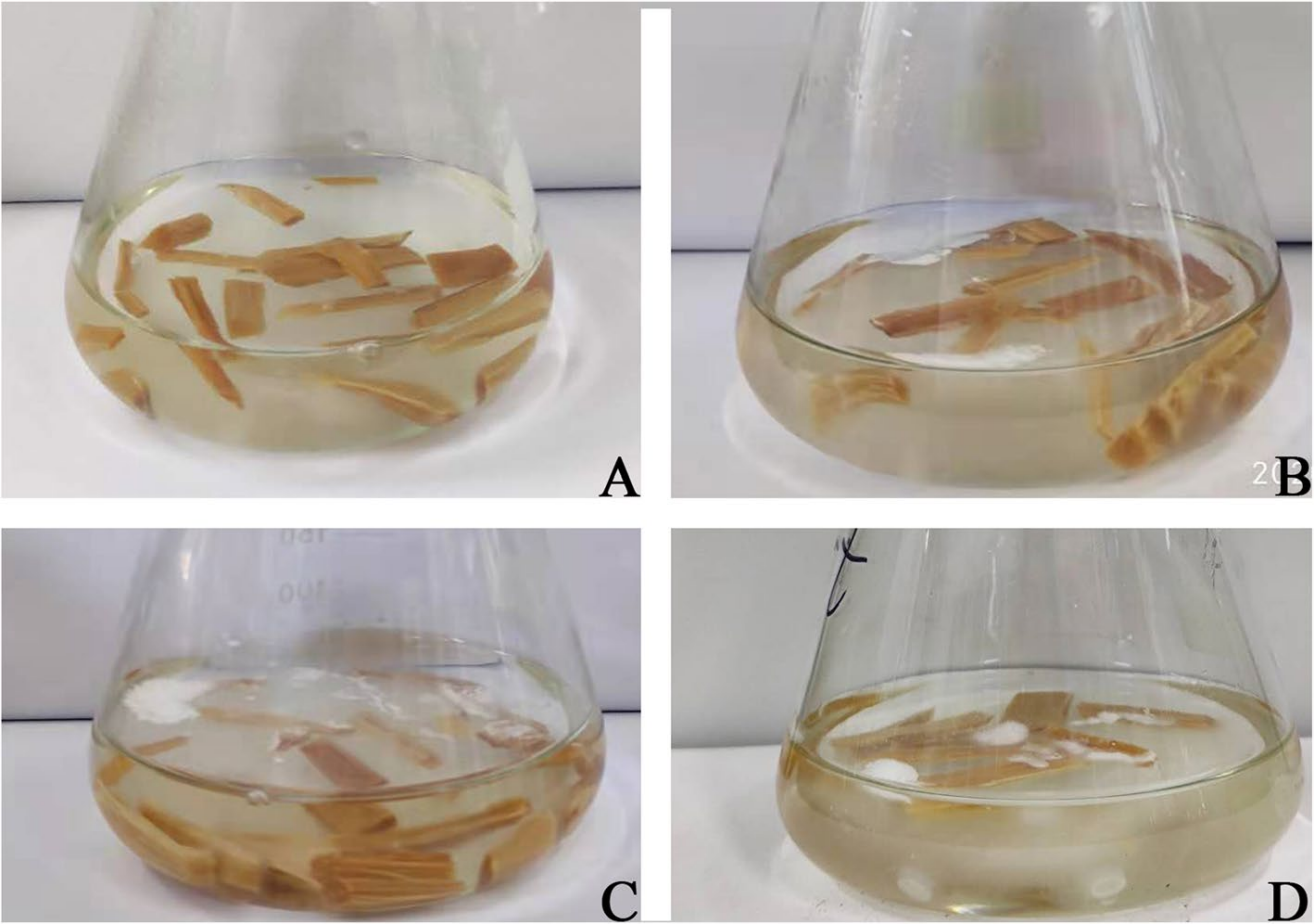
Mycelium growth at different times. **A** 3 days, **B** 5 days, **C** 7 days, **D** 11 days

**Fig. 3:**
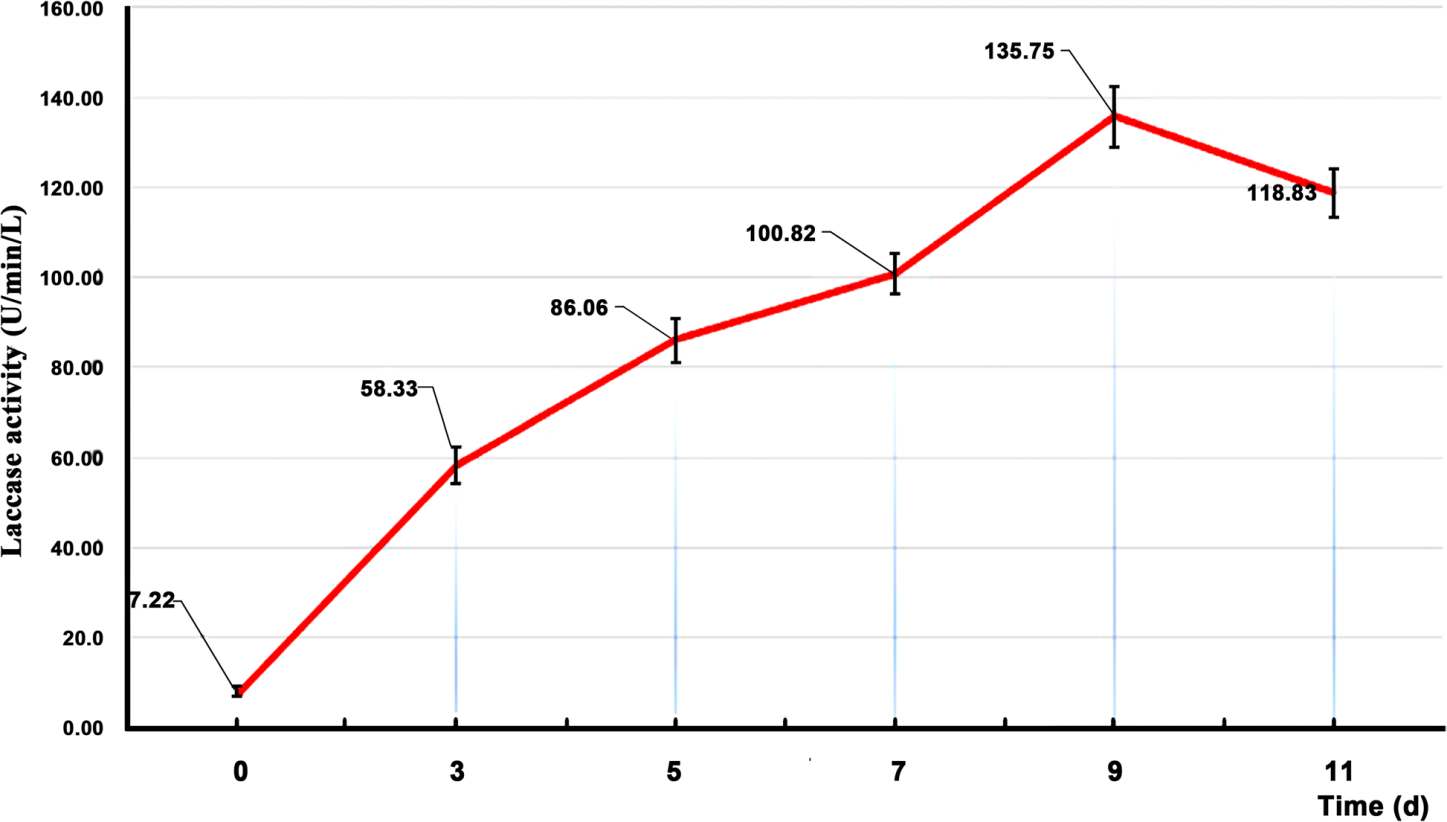
Laccase activity in LNAS at different times

Laccase activity increased with time before the 9d. The peak value reached 135.75 U/min/L. After the 9th day, laccase activity began to decrease ((Fig. 2 and 3).

### RNA sequencing data and DEGs

After 0, 3, 5, 7, and 11 days, total RNA was extracted by mycelia, and 114.55 Gb of cleaning data were obtained. A total of 15 libraries were constructed, named CK 1–3 (0 days), MX11-3 (3 days), MX2 1–3 (5 days), MX3 1–3 (7 days), and MX4 1–3 (11 days). The percentage Q score of Q30 bases was at least 91.84%. The reference genome of this species is Tragib1 in the Joint Genome Institute (JGI) database. The comparison efficiency of the sample data of all 15 transcriptional groups was between 88.51% and 91.37% (Table S2). After transcript and genome alignment, a total of 12,885 genes were identified. The top two PCA components (PC1: 43.1% and PC2: 10.8%,) distinguished well with a clear spatial pattern in this study (Fig. S1). A total of 5,232 DEGs were identified in different timepoint groups, including 1,803 up-regulated genes and 3,429 down-regulated genes (Table S3).

GO classification analysis demonstrated that the DEGs were distributed in 38 categories of biological process, cellular component, and molecular function. Biological process categories were mainly related to cellular processes, metabolic processes, single-organism processes, biological regulation, and response to the stimulus. Catalytic activity had a large number of DEGs in the molecular function (Table S4). GO enrichment analysis (p ≤ 0.05) of DEGs between identified, including 85 (0 vs 3 days), 135 (3 vs 5 days), 132 (5 vs 7 days), and 132 (7 vs 11 days). Specifically, the lignin catabolic process (GO: 0046274), phenylpropanoid catabolic process (GO:0046271), and lignin metabolic process (GO:0009808) were enriched in four adjacent time points groups. The mycelium grew from the adaptation period (0—3 days) to the rapid growth period (3—5 days) and tended to be stable at last (5—11 days). In the ninth column, there were 11 go terms. Some of them are flavin adenine dinucleotide binding (GO:0050660), transition metal ion transport (GO:0000041), heme binding (GO:0020037), ubiquitin binding (GO:0043130), ER-associated ubiquitin-dependent protein catabolic process (GO:0030433) and ergosterol biosynthetic process (GO:0006696) distributed in 10 comparison groups. Ergosterol affects the absorption and utilization of nutrients by affecting the activity of membrane-bound ATP enzymes and regulating the fluidity of cell membranes in fungi. As one of the small molecular proteins, ubiquitin can degrade Intracellular proteins which play a very important role in *T. gibbosa* response wood (Fig. S2).

DEGs and hub genes were randomly selected for qRT-PCR validation. The fold changes in the levels of these genes were consistent with the data of RNA_seq and qRT-PCR (Fig. S3A). Thus, qRT-PCR analysis confirmed that the changes detected by the RNA sequencing analysis were reliable (*R*^2^ = 0.81) (Fig. S3B).

### Weighted gene co-expression network analysis

Finally, 12,329 genes were selected for WGCNA. The scale-free network obtained by power processing at β = 22 resulted in an adequate fit with R^2^ = 0.85 (> 0.8) with average connectivity approaching 0. Therefore, β = 22 was used to construct the scale-free network (Fig. S7). The dynamic clipping algorithm was used to cluster and divide genes into modules. The modules were clustered based on the ME distance. Modules with high K.I. values were placed at the top of the branch, which had the highest interconnection with the other modules. The horizontal line represented the threshold used to define the meta-module (Fig. S4). A combination of similar modules yielded a total of 24 gene co-expression modules marked with colors. These modules contained from 45 to 3,154 genes (Fig. S8). The correlations between 24 modules based on the heatmaps and clustering according to the expression of the genes were used to quantify the co-expression similarity of all modules (Fig. S5).

**Fig. 4:**
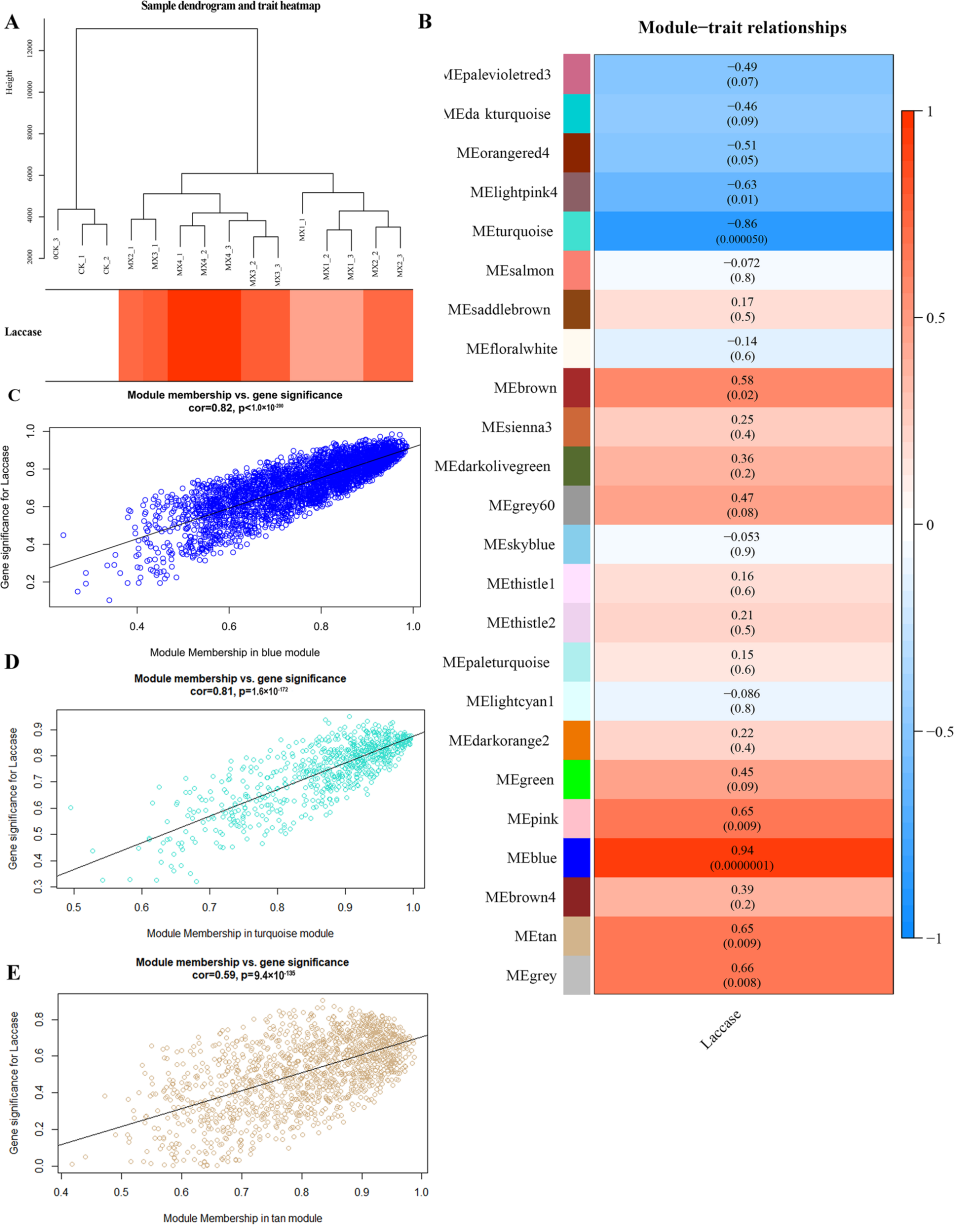
Correlation diagrams of laccase activity. **A** Sample dendrogram and trait heatmap; **B** Module-trait; **C** Module membership vs gene significance in the blue module; **D** Module membership vs gene significance in the turquoise module; **E.** Module membership vs. gene significance in the tan module (each dot represents a single gene in each module; the y-axis corresponds to GM, and the x-axis corresponds to MM; graphs show the regression line, related values and *p* values)

**Fig. 5:**
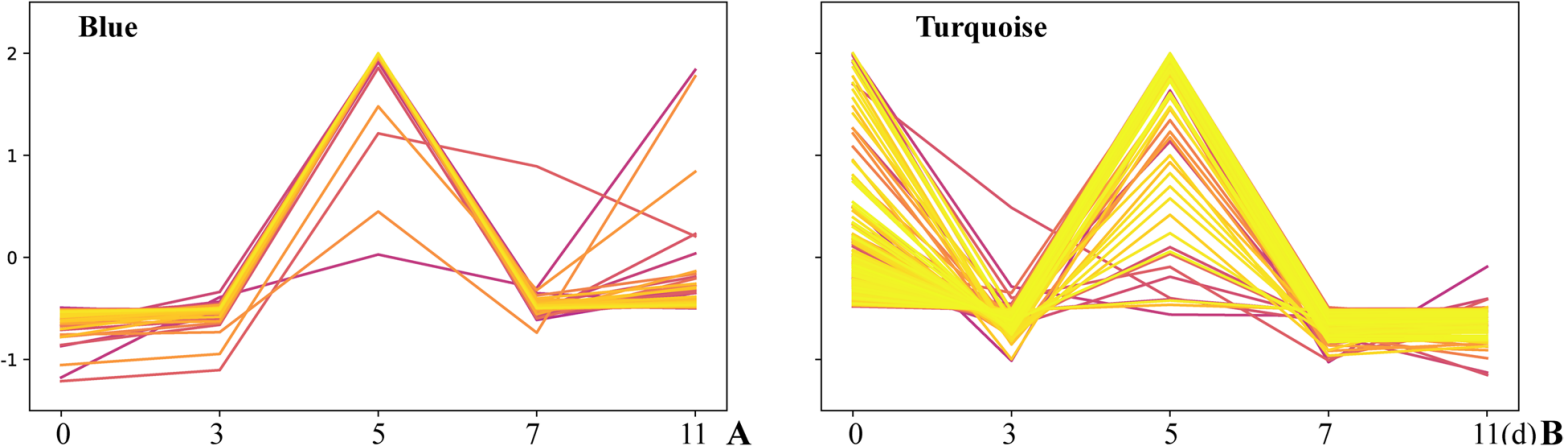
The gene expression patterns in two module. **A** Blue module; **B** Turquoise module

MEs are the first principal component of a given module and can be regarded as representative of the gene expression profile of a module. Analysis of the changes in laccase activity included 15 samples that were used to construct a map of correlations between the samples and laccase activity (Fig. 4A). The relationships between each module and laccase activity were evaluated based on significant correlations (Fig. 4B). The results showed that 6 modules had highly specific correlations with laccase activity (|r|> 0.60 and *p* < 0.001), including turquoise, lightpink4, tan, pink, grey, and blue module. Laccase activity was the most negatively correlated with the turquoise module (r = -0.86, *p* = 5 × 10^–5^) and positively correlated with the blue module (r = 0.94, *p* =  × 10^–7^). Genetic compositions of specific modules were used to investigate the network-specific properties of gene significance (GS) and module membership (MM) by calculating the ME values of a single module and the corresponding genes. Higher content of GS and MM of a gene indicated that characteristics of this gene are more impactful [36]. Therefore, higher R-values and smaller p-values of a particular module indicated that the module members were more representative of the module characteristics. The blue (cor = 0.82, *p* < 1 × 10^–200^) (Fig. 4C), turquoise (cor = 0.81, *p* = 1.6 × 10^–172^) (Fig. 4D) and tan modules (cor = 0.59, *p* = 9.4 × 10^–135^) (Fig. 4E) may play a more important biological role in the processes related to laccase activity compared to that of other modules. The blue module and turquoise module are used for the following analysis. The trend of gene expression was the same in the two modules. Both modules reached a peak on the fifth day and fluctuated in the later stage of response to wood. The gene expression was stable for 0–3 days in the blue module. In the turquoise module, gene expression decreased rapidly in the early stage of response to wood (Fig. 5).

### Constructing the gene expression network based on hub genes in the module

**Fig. 6:**
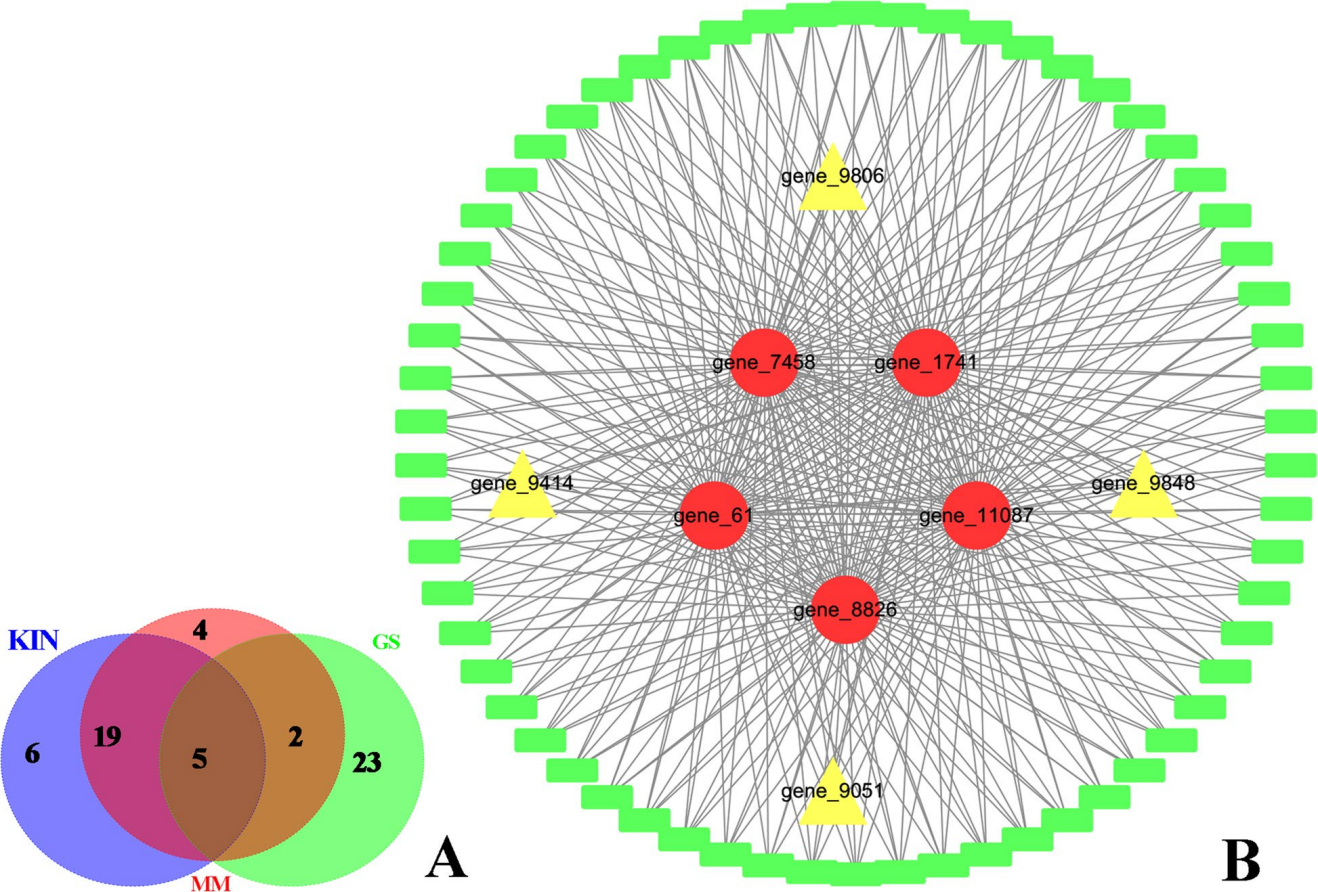
Characterization of the blue module. **A** Venn diagram of the top 30 genes of the blue module based on high gene significance (GS), high module membership (MM) and high intramodular connectivity (K.in); **B** Interaction of the gene co-expression patterns of 66 highly relevant genes in the blue module is shown as a network constructed using Cytoscape 3.7.1. Note: Red markers indicate the key hub genes, and yellow markers indicate the transcription factors

In blue module, 1,329 genes were selected with GS > 0.8, *p* < 0.01 and |MM.blue|> 0.8. The top 30 genes with high GS, MM, and K. in values, among which 5 genes overlap as hub genes (Fig. 6A and Table S5). The ranges of the values for each gene in the blue module network based on these three parameters (threshold = 0.02) were as follows: K.in count (4.72 ~ 183.70; average 63.80); GS score (0.80 ~ 0.98; average 0.86); and MM count (-0.96 ~ 0.99; average 0.78) (Fig. S6). In especial, 66 genes were highly linked to the hub genes in the network. Specifically, there were 4 transcription factors, including gene_9051, gene_9414, gene_9848, and gene_9806, that are linked to 66 highly relevant genes (Fig. 6B).

**Fig. 7:**
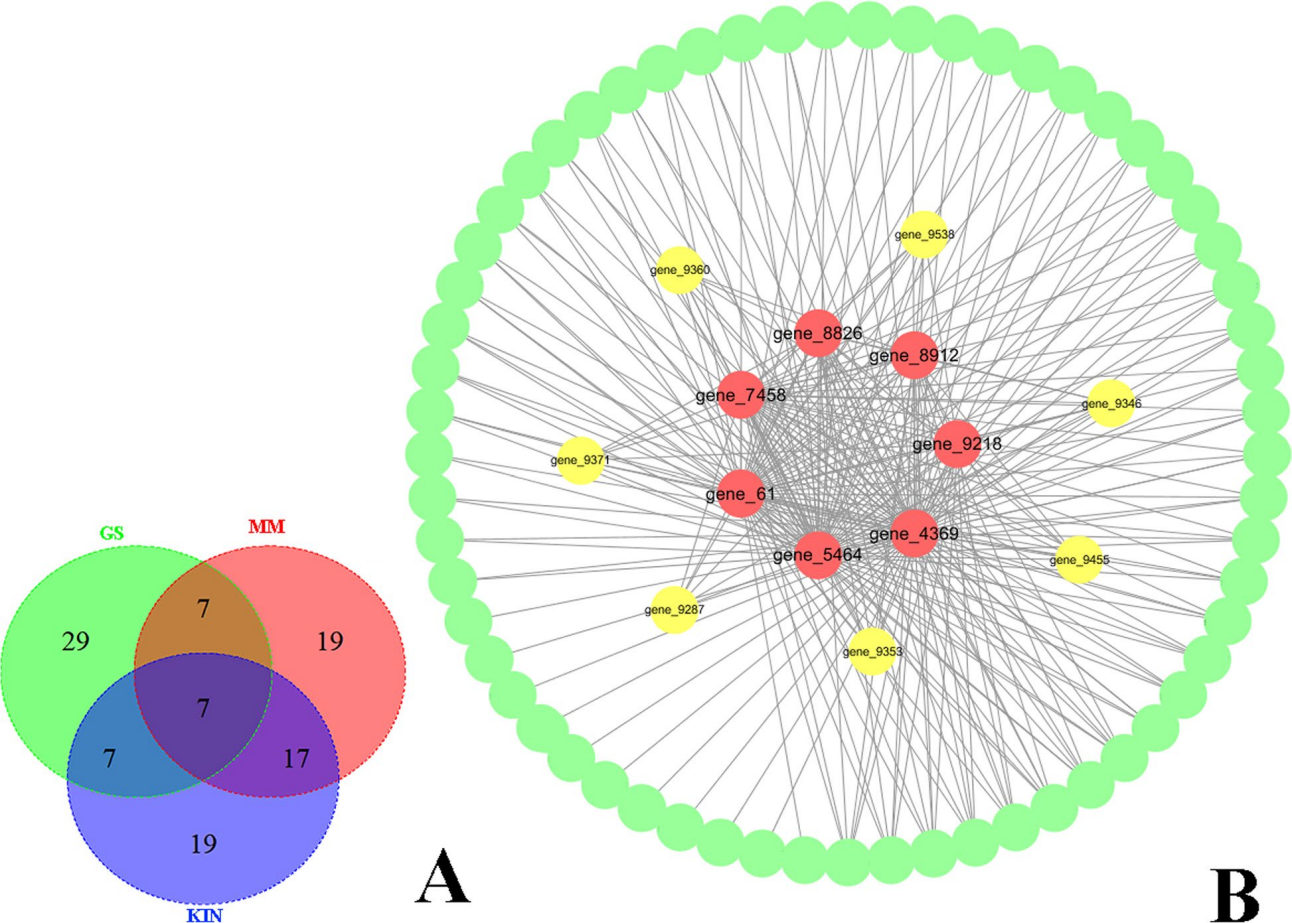
Characterization of the turquoise module. **A** Venn diagram of the top 50 genes of the turquoise module based on high gene significance (GS), high module membership (MM), and high intramodular connectivity (K.in); **B** Interaction of the gene coexpression patterns in the turquoise module; 75 genes with the highest weight in the turquoise module were used to construct a network by Cytoscape 3.7.1. Note: red markers indicate the key hub genes, and yellow markers indicate the genes with high correlations

In the turquoise module, the top50 genes of interest in the turquoise module with high GS, MM, and K.in values were shown in the Venn map that detected 7 genes overlapping, as hub genes (Fig. 7A). The range of the values for each gene in the turquoise module network based on three parameters was as follows: K.in count (12.57 ~ 169.98; average 72.48); GS score (0.90 ~ 0.98; average 0.92); and MM count (-0.96 ~ 0.97; average -0.77) (Fig. 7B and Table S6). Moreover, seven genes were highly linked to the central genes in the network (Fig. 7B).

### Functional analysis of the blue and turquoise modules

**Fig. 8:**
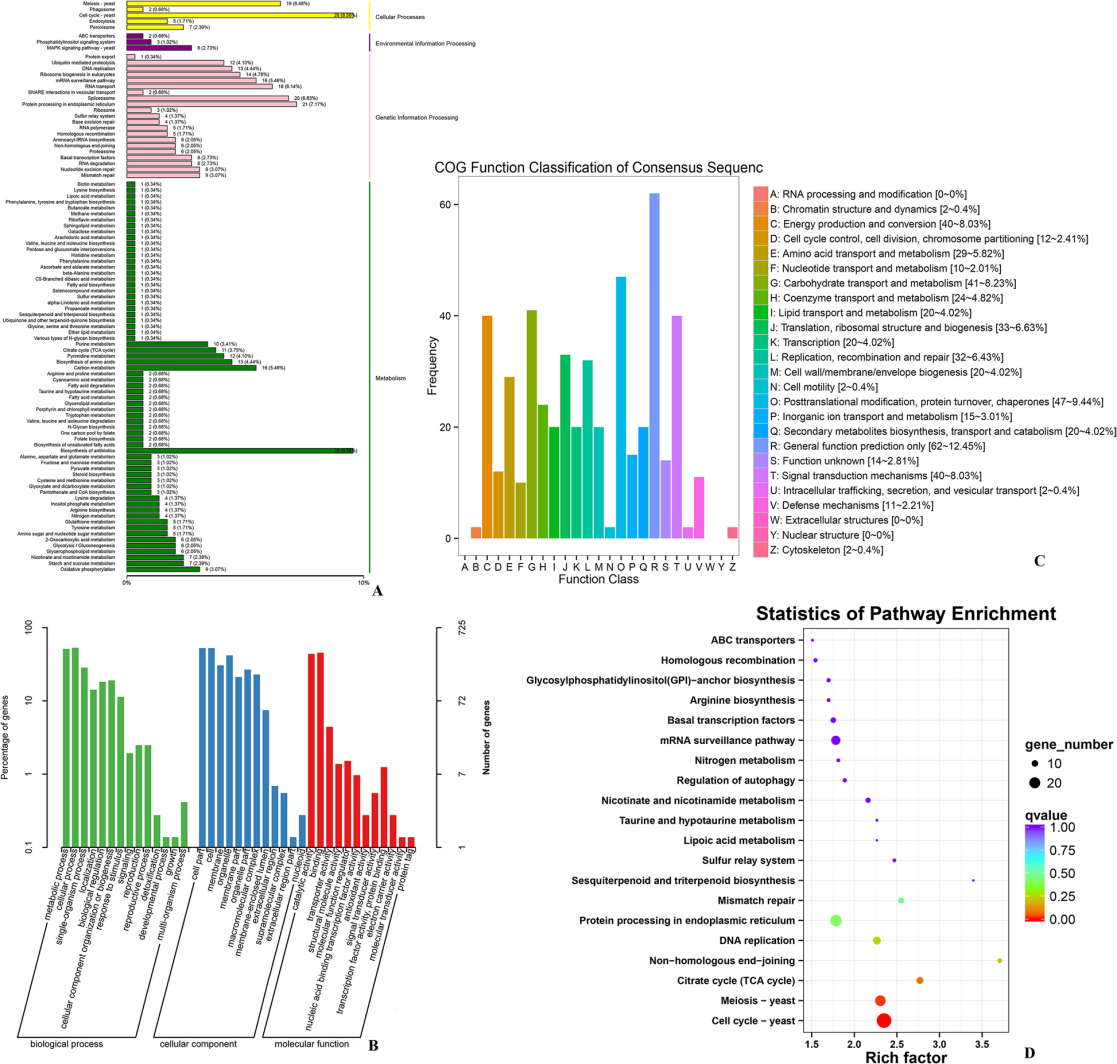
Feature analysis of the blue module. **A** KEGG pathway classification of the hub genes of the module; **B** GO annotation of the hub genes of the module; **C** COG annotation of the hub genes of the module; **D** KEGG pathway enrichment of the hub genes of the module

To better understand the gene function of these two modules in laccase secretion, we analyzed the genes function in the blue and turquoise modules. A total of 444 hub genes in the blue module were annotated for COG classification, and the classes with the most annotations were as follows: Q: Secondary metabolites biosynthesis, transport, and catabolism; H: Coenzyme transport and metabolism; E: Amino acid transport and metabolism; T: Signal transduction mechanisms; G: Carbohydrate transport and metabolism; and O: Posttranslational modification, protein turnover, chaperones (Fig. 8C). A total of 725 genes were annotated and enriched in 73 GO terms (belonging to biological process, cellular component and molecular function; *p* < 0.05). Most of the genes were enriched in protein refolding, ER-associated ubiquitin-dependent protein catabolic process, cell cycle process, and sterol metabolic process of the biological process category. Enrichment in the cellular component category included nuclear part, mitochondrial outer membrane, and site of the double-strand break. In the molecular function category, enrichment included RNA binding, ATP binding, and succinate dehydrogenase activity (Table S7 and Fig. 8B). A total of 554 genes were annotated and enriched in KEGG terms, mainly including cell cycle, meiosis, citrate cycle (TCA cycle), non-homologous end-joining, DNA replication, protein processing in the endoplasmic reticulum and nicotinate and nicotinamide metabolism (ko00760) (Table S8 and S9, Fig. 8 A and D). 74 transcription factors in 44 families were found in the blue module (Table S10).

**Fig. 9:**
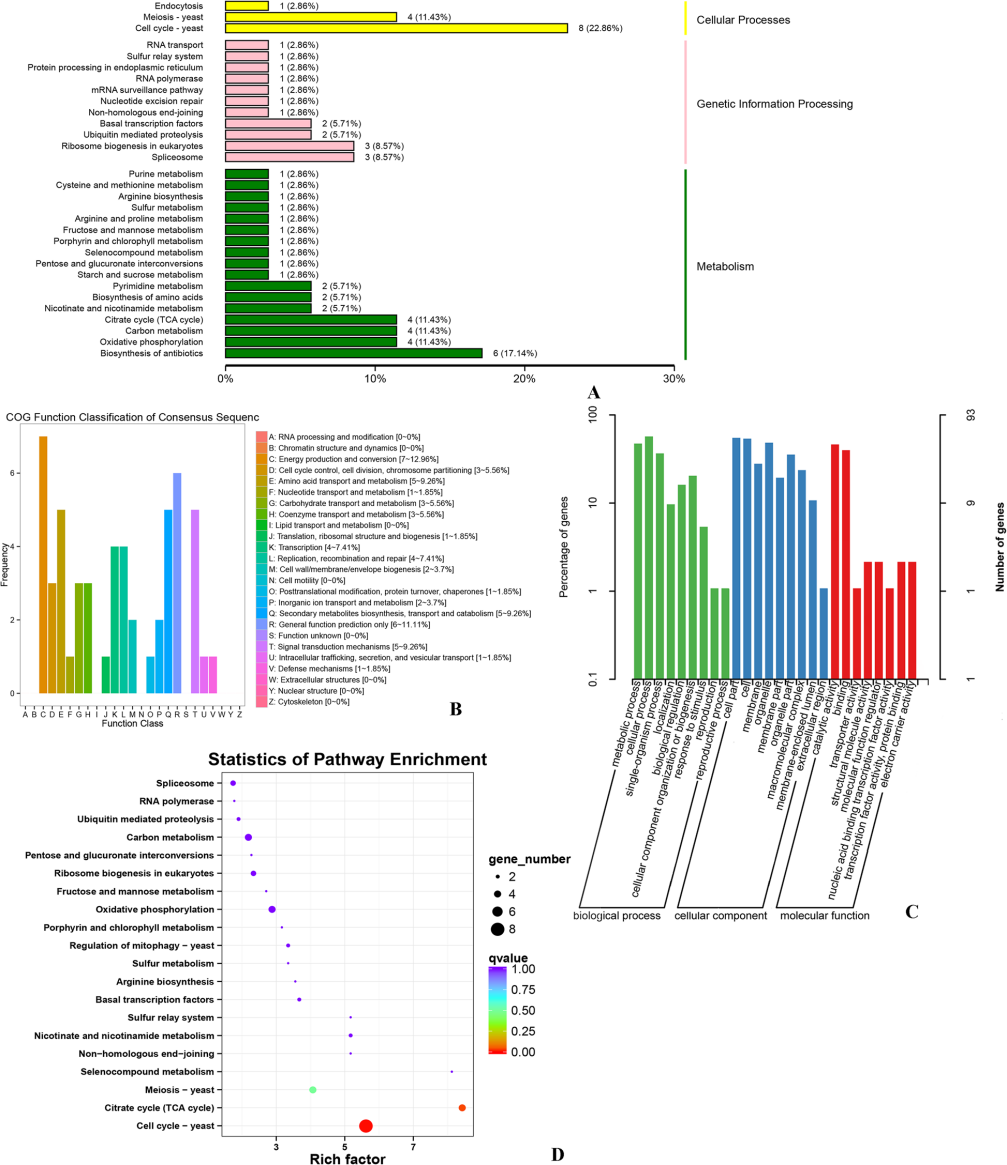
Feature analysis of the turquoise module. **A** KEGG pathway classification of the hub genes of the module; **B** COG annotation of the hub genes of the module; **C** GO annotation of the hub genes of the module; **D** KEGG pathway enrichment of the hub genes of the module

A total of 57 genes in the turquoise module were annotated from the COG database. C: Energy production and conversion; T: Signal transduction mechanisms; E: Amino acid transport and metabolism; and Q: Secondary metabolites biosynthesis, transport, and catabolism were classified (Fig. 9B). A total of 93 genes were annotated and enriched in 47 GO terms; the majority of the genes were enriched in the biological process category, including protein targeting, the establishment of protein localization to organelle, proteolysis involved in cellular protein catabolic process, modification-dependent protein catabolic process, mitochondrion localization, and ubiquitin-dependent protein catabolic process(Table S11, Fig. 9C). 72 genes were annotated and enriched from KEGG database, mainly including cellular processes, genetic information processing, and metabolism pathway. The main terms specifically included cell cycle, citrate cycle (TCA cycle), meiosis, oxidative phosphorylation, nicotinate, nicotinamide metabolism, and carbon metabolism (Table S12 and S13, Fig. 9A and D). Seven transcription factors in seven families were predicted in the turquoise module (Table S14).

## Discussion

RNA-seq data can comprehensively and quickly obtain almost all sequence and expression information of a specific cell or tissue in a certain state. We examined RNA-Seq data of *T. gibbose* under wood chips at five-time points. Then, the gene network is constructed by using the WGCNA. KEGG pathway and GO enrichment analysis were performed on biologically significant modules and identified key central genes in the module. Understanding the synthetic pathway of laccase activity can be used to increase the lignin degradation rate. It will be used for clean bio-energy, industrial production, and environmental protection.

Time-series gene expression data provide the trajectories of the gene expression levels over time to monitor the continuity of all events by identifying DEGs at multiple time points and assessing the enrichment of biological processes. The following common GO terms were enriched in DEGs between adjacent time points: transition metal ion transport (GO:0000041), phenylpropanoid catabolic process (GO:0046271), lignin catabolic process (GO:0046274), lignin metabolic process (GO:0009808), flavin adenine dinucleotide binding (GO:0050660), antioxidant activity (GO:0016209) and oxidoreductase activity, acting on the aldehyde or oxo group of donors, NAD or NADP as acceptor (GO:0016620). White-rot fungi are known to secrete extracellular enzymes (mainly laccase, manganese peroxidase, and lignin peroxidase to degrade lignin. These enzymes require the transport of metal ions: Cu, Zn, Fe, and Mn [27]. Additionally, coenzymes are compounds with special chemical structures and functions, which participate in enzyme-catalyzed redox reactions and transfer electrons, atoms, or groups in enzyme-catalyzed reactions; for example, NAD^+^ or NADP^+^ can be reduced by hydride ion. The redox reaction is the main process of lignin degradation, indicating that wood chips have been degraded. On day 3, the number of DEGs was significantly decreased, indicating early adaptation of the fungus to wood chips. At this time point, the gene expression of cytochrome P450, glutathione S-transferases (GSTs), and ABC transporters were significantly downregulated, indicating that compounds released from the biomass or newly produced in the process of wood chip degradation are related to active detoxification (Table S15) [38, 39].DEGs included a downregulated laccase-3, an upregulated laccase-1, and 4 upregulated and 6 downregulated glycosyl hydrolases of various families (Table S15). The results showed that the start of laccase production induced sugar group decomposition. The citrate cycle (TCA cycle) (ko00020) included 6 downregulated genes; galactose metabolism (ko00052) included 5 downregulated genes, and glycolysis/gluconeogenesis (ko00010) included 10 downregulated genes that were significantly enriched (*p* < 0.05), indicating that the TCA cycle, galactose metabolism, and glycolysis/gluconeogenesis were inhibited and that the expression of pyruvate kinase was also inhibited. Specifically, carbon metabolism (ko01200) included 43 downregulated DEGs and an upregulated DEG. Lignin supplied by wood is a cyclic phenol compound, and phenol compounds can be degraded through the tyrosine metabolism pathway (ko00350) to produce simple sugars. Three FAD-binding domain-containing genes were upregulated. Flavin adenine dinucleotide (FAD) activates peroxidase by producing H_2_O_2_ [40] and promotes lignocellulose decomposition by regenerating the activity of lytic polysaccharide monooxygenase (LPMO) towards cellulose via the redox cycle of phenols and the corresponding quinones in the media [41]. Fungal growth is well-known to require a carbon source (Fig. 6). In the early adaptation stage, the demand for carbon sources is relatively low. However, inhibition of carbon metabolism and the TCA cycle indicated that the metabolism of energy mainly depended on some other oxidoreductases, such as laccase, tyrosine kinase, and glycosyl hydrolase, to decompose extracellular proteins and wood chips to provide energy. Stimulation of white-rot fungus *Phanerochaete chrysosporium* with exogenous benzoic acid induced a dramatic and coordinated decrease in the levels of most organic acids of the TCA cycle, including citrate, cis-aconitate, 2-oxoglutarate, fumarate, and malate. The accumulation of most amino acids and some fatty acids may be related to a decrease in the levels of TCA intermediates [42] similar to the results obtained in the present study. The expression of the genes related to aspartyl protease, glutamine amidotransferase, and serine/threonine kinase was upregulated. Aspartic acid is the raw material for the indirect synthesis of isoleucine, threonine, methionine, lysine, and other amino acids, and amidotransferase is one of the key enzymes in the indirect synthesis of glutamyl-tRNA [43]. Degradation of protein antigens and stimulation of the activity of other enzymes is the main function of aspartic protease, suggesting that the basic pathway of amino acid synthesis is downregulated, and the synthesis of amino acids through the indirect pathway is upregulated, which is beneficial for enhanced adaptation of the strain to the changes in nutritional conditions. Serine/threonine protein kinase (S/TK) is involved in protein phosphorylation [44]. *Trichoderma reesei* and *Neurospora crassa* have been shown to catalyze serine and threonine phosphorylation to transfer the extracellular signals to the intracellular space to subsequently promote or inhibit cellulase secretion by affecting downstream gene transcription or protein modification [45–47]. The mycelium began to proliferate after 3–5 days and was in the exponential stage. After 5 days, the number of DEGs decreased; the mycelium growth reached a plateau, and vital activity was essentially stable.

These genes play a role in the adaptation of the fungi to lignin decomposition [48]. Cytochrome P450 mediated pentachlorophenol oxidation [49]; the CYP512, CYP5136, and CYP5150 families can oxidize a wide range of hydrocarbons, including phytochemicals and key metabolic intermediates in fungi, such as steroids, indicating that these families play a role in the primary and secondary metabolism in fungi [50]. Matsuzaki et al. studied the intracellular changes in *P. chrysosporium* during benzoic acid metabolism at the proteome and metabolic levels and found aryl alcohol dehydrogenase, aromatic aldehyde dehydrogenase, and cytochrome P450 play the key roles in benzoic acid metabolism [42]. In the presence of lignin, the microsomal proteins of the RP-78 strain of *P. chrysosporium* were expressed, and transport proteins and cytochrome P450 were involved in the degradation of lignin [51]. In the secondary metabolism, lignin degradation was dominated by oxidoreductases, such as laccase, which were steadily increased, and cytochrome P450 and ABC transporters were initially inhibited; manganese peroxidase began to appear at longer fermentation times. Cytochrome P450 and ABC transporters were activated by the accumulation of lignin intermediates. The results were used to investigate how wood treatment of *T. gibbosa* induces the coordination of these enzymes with each other to degrade lignin and what secondary metabolic pathways play the key roles in this process (Table S15).

Many studies investigated the expression of the genes related to laccase; however, little attention has been paid to the analysis of the laccase activity of the complex co-expression networks. The present study reports the results of the WGCNA of wood treatment that identified 24 co-expression network modules. The turquoise, light pink 4, tan, pink, grey, and blue modules were significantly correlated with laccase in *T. gibbosa* (|r|> 0.60 and *p* < 0.001). Additionally, the conservation of two modules in various datasets was studied in detail. WGCNA of the meta-module and additional analysis of the network characteristics (GS, MM, and K.in) confirmed that the blue and turquoise modules were the main modules. GO classification entries were highly consistent and mainly concentrated in the following categories: biological processes: metabolic processes; cellular processes: cellular components, cell parts, and cell; and molecular function: binding and catalytic activity. These data are consistent with the pathway of laccase production in the cells. Enrichment data (*p* < 0.05) indicated the presence of the following terms in two modules: cellular component organization (GO:0016043), mitochondrial outer membrane (GO:0005741), RNA binding (GO:0003723), succinate dehydrogenase activity (GO:0000104), succinate dehydrogenase (ubiquinone) activity (GO:0008177), carboxypeptidase activity (GO:0004180), exopeptidase activity (GO:0008238), flavin adenine dinucleotide binding (GO:0050660), oxidoreductase activity, acting on the CH-CH group of donors, quinone or related compound as acceptor (GO:0016635) and translation factor activity, nucleic acid binding (GO:0008135) (Additional file 12 and 16). The results showed that these terms were related to the secretion of laccase or corresponded to the genes which were co-expressed in the mycelium to perform vital activities as a combination. The mitochondria were very active. Succinate dehydrogenase is an enzyme of the mitochondrial inner membrane that binds to cytochrome oxidase and is one of the hubs connecting oxidative phosphorylation and electron transport. Succinate dehydrogenase is the only multisubunit enzyme of the TCA cycle integrated into the membrane. Flavin adenine dinucleotide (FAD) is the prosthetic group of succinate dehydrogenase. FAD binds to the histidine residue of the enzyme via a covalent bond. Laccase oxidation transfers electrons from phenolic substrate molecules to form semiquinone radicals, and other oxidoreductases acting on the CH-CH group are involved in the degradation of phenols. Exopeptidase and carboxypeptidase are proteolytic enzymes that catalyze the hydrolysis of the peptide bond at the end of the polypeptide chain and free terminal amino acid. The KEGG pathways of the blue and turquoise modules were also highly consistent. In particular, the pathway analysis of these modules showed that Cell cycle-yeast (ko04111), Citrate cycle (TCA cycle) (ko00020), Meiosis – yeast (ko04113), and Nicotinate and nicotinamide metabolism (ko00760) were the core pathways of the blue and turquoise modules linked to the secretion of laccase. Oxidoreductase activity, acting on the CH-NH_2,_ NAD, or NADP as acceptor (GO:0016639) and glutamate dehydrogenase (NAD^+^) activity (GO:0004352) were present only in the blue module. Oxidoreductase activity, acting on the aldehyde or oxo group of donors (GO:0016903) was present only in the turquoise module. Nicotinate and nicotinamide metabolism were closely related to laccase activity. However, few studies have reported the effects of nicotinate and nicotinamide metabolism on laccase activity. Therefore, relationships between nicotinate and nicotinamide metabolism with laccase activity remain to be verified.

**Fig. 10:**
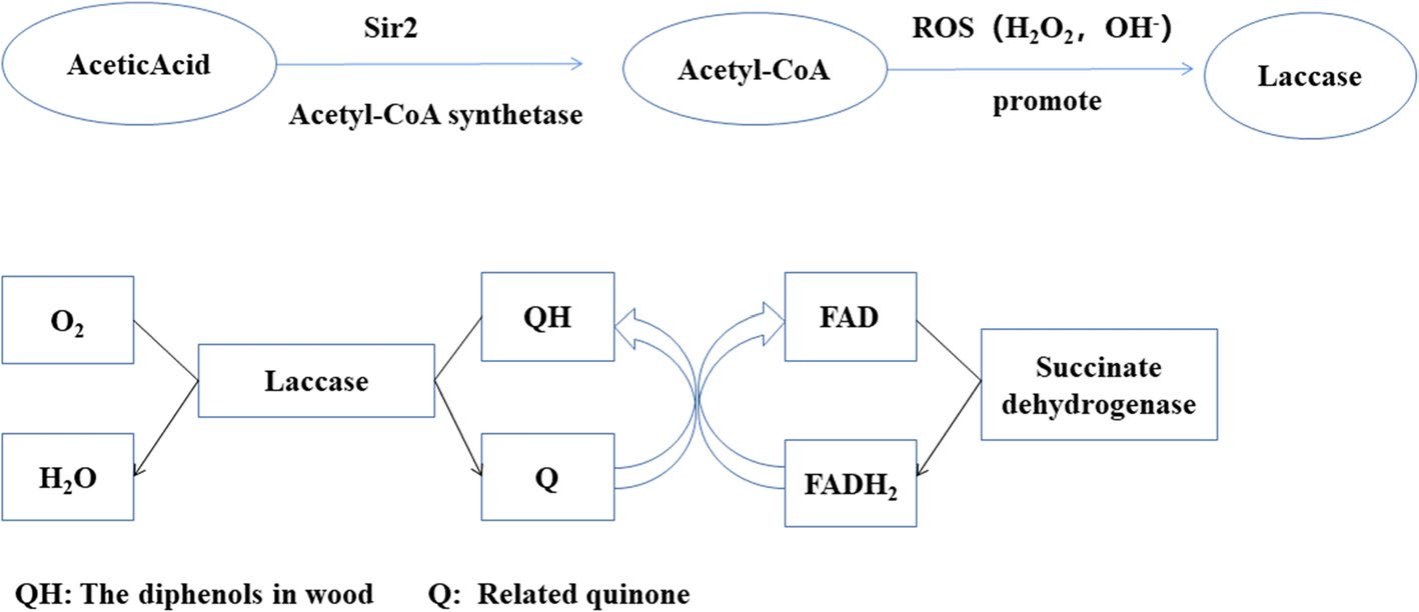
The correlations between mitochondrial metabolism and laccase activity

Extensive cross-validation of the blue module based on the MM, GS and K.in index identified top network hub genes: gene_8826 (MW199767) (jgi.p|Tragib1|1387922), gene_7458 (MW199766) (jgi.p|Tragib1|1323325), gene_61 (MW199765) (jgi.p|Tragib1|1414099), gene_1741 (MH257605) (jgi.p|Tragib1|1311301) and gene_11087 (MK805159) (jgi.p|Tragib1|1400843) (Fig. 8A and Table 1). Gene_7458 encodes a protein involved in the maintenance of mitochondrial morphology that is related to the phospholipid transport through the mitochondrial outer membrane, participates in the assembly of mitochondrial outer membrane translocase complex, and transports mitochondrial amino acids to the endoplasmic reticulum to synthesize laccase. Gene_1741 encodes laccase-3 and is the key gene regulating laccase synthesis. Gene_61 encodes a protein of the sir2 family of NAD-dependent protein deacetylases. Several studies investigated sir2 in animals and plants; however, the function of fungal sir2 was studied in detail only in yeast. Yeast sir2 is related to senescence and oxidative stress (Fig. 8). Sir2 is the key enzyme of the deacetylation and gluconeogenesis pathways involving the hydrolysis of NAD^+^ products, resulting in the loss of the catalytic activity of sir2 [52–54] to reduce intracellular sugar storage and delay cell senescence. Hydrolysate of lignocellulose is considered to be a renewable raw material for microbial fermentation [55]. Acetic acid is present at a very high concentration in these hydrolysates and is a powerful inhibitor of microbial growth and fermentation. Sir2 enhanced the activity of acetyl-CoA synthetase and converted acetic acid to acetyl-CoA [56]. Sir2 was predicted to promote the synthesis of acetyl-CoA, resulting in excessive production of reactive oxygen species (ROS), oxidative stress, and a series of effects on the regulation of the cell cycle, apoptosis, and autophagy [57, 58]. On the other hand, low concentrations of reactive oxygen species (H_2_O_2_ and OH^−^) promoted the production of laccase by white-rot fungus, and high concentrations of ROS inhibited their production (Fig. 10).

**Table 1:** Annotations of genes of interest in two modules

Module	Gene ID	Gene_name	Pfam_annotation	Swiss_Prot_annotation
Turquoise	gene_61	jgi.p|Tragib1|1,414,099	Sir2 family	NAD-dependent protein deacetylase hst4
	gene_8826	jgi.p|Tragib1|1,387,922	SKIP/SNW domain	Pre-mRNA-processing protein 45
	gene_4369	jgi.p|Tragib1|1,316,018	DNA-directed RNA polymerase III subunit Rpc31	–
	gene_8912	jgi.p|Tragib1|1,326,595	IPP transferase	tRNA dimethylallyltransferase
	gene_5464	jgi.p|Tragib1|1,418,634	CybS, succinate dehydrogenase cytochrome B small subunit	Succinate dehydrogenase [ubiquinone] cytochrome b small subunit, mitochondrial
	gene_9218	jgi.p|Tragib1|1,421,912	–	Adenylate-forming reductase 03,009
	gene_7458	jgi.p|Tragib1|1,323,325	Maintenance of mitochondrial morphology protein 1	Maintenance of mitochondrial morphology protein 1
Blue	gene_8826	jgi.p|Tragib1|1,387,922	SKIP/SNW domain	Pre-mRNA-processing protein 45
	gene_7458	jgi.p|Tragib1|1,323,325	Maintenance of mitochondrial morphology protein 1	Maintenance of mitochondrial morphology protein 1
	gene_61	jgi.p|Tragib1|1,414,099	Sir2 family	NAD-dependent protein deacetylase hst4
	gene_1741	jgi.p|Tragib1|1,311,301	Multicopper oxidase	Laccase-3
	gene_11087	jgi.p|Tragib1|1,400,843	SNF2 family N-terminal domain; Zinc finger, C3HC4 type	Uncharacterized ATP-dependent helicase C23E6.02

Interestingly, gene_11087 was predicted to encode a transcription factor, and Pfam database comparison identified an SNF2 family N-terminal domain (7.3e-61), helicase conserved C-terminal domain (3.1e-18), and zinc finger (C3HC4) (0.00015) in this protein; however, the function of this protein is unclear. Annotation analysis indicated that this protein can be related to ATP binding (GO:0005524) and zinc ion binding (GO:0008270). Snf2 family proteins can influence gene expression by using the energy of ATP hydrolysis to change the interactions between histones and DNA; most of the Snf2 proteins are related to the chromatin remodeling complexes, which are likely to change the activity of the core ATP enzymes [59, 60]. However, a protein encoded by gene_11087 (jgi. p|Tragib1|1400843) has an SNF2 family domain, and it is not known whether this protein acts on ATP. It is not known whether this gene is related to the expression of laccase, which requires further exploration. The co-expression patterns of the genes of the blue module and their regulatory factors (TF) were used to predict a total of 74 genes from 43 families (Table S10). Prediction using 66 highly linked genes identified 4 transcription factors. Gene_9051 (jgi.p|Tragib1|1327035), gene_9414 (jgi.p|Tragib1|1542416), gene_9848 (jgi.p|Tragib1|1393729) and gene_9806 (jgi.p|Tragib1|1422389) belong to the AGC-Pl, C2H2, CAMK_AMPK and IWS1 families (Table S10), which may regulate the key circuits involved in the process of laccase secretion. In addition to the hub genes in the turquoise module, gene_5464 was identified as another specific gene. This gene encodes a mitochondrial inner membrane protein, succinate dehydrogenase cytochrome B small subunit (Table 1), that participates in the TCA cycle. The metabolic transfer activates redox metabolic systems, such as lignin, laccase, manganese peroxidase, and cytochrome P450 [42].

Optimization of the fungal lignin degradation system enhanced the extracellular enzyme system, and a comprehensive design of the metabolic structure is needed. Therefore, we suggest that these key central genes are very important for laccase activity and deserve further attention. Understanding the mechanism of *T. gibbosa* laccase production and enhancing the laccase activity of *T. gibbosa* will assist with applications based on an increase in the rate of lignin degradation by *T. gibbosa*.

## Supplementary Information

**Additional file 1: Fig. S1.** Principal components analysis of variation and correlation coefficient analysis. **Fig. S2.** The upset plot shows the enrichment in DEGs in each group (*P*_value<0.05). Set Size represents the number of go terms. **Fig. S3.** Quantitative real-time polymerase chain reaction. A. The gene expression of qRT-PCR and RNA_seq. The short line represents the standard deviations on the column in qRT-PCR. B. Gene expression correlation between RNA-Seq and qRT-PCR data. Each blue dot indicates the selected genes. **Fig. S4.** Clustering of module eigengenes. **Fig. S5.** Modules significance correlation. **Fig. S6.** Interaction of co-expression patterns of the genes in the blue module. **Fig. S7.** Selection of the soft-thresholding power. A. The panel shows the scale-free fit index versus soft-thresholding power. B. The left panel displays the mean connectivity versus soft-thresholding power. C. Verification of the memory network using the selected values. R_2_=0.85. **Fig. S8.** Clustering dendrograms of detected genes and modules. Note: different colors represent different modules. **Table S1.** Primers used in the present study. **Table S2.** Transcriptome sequencing data statistics. **Table S3.** Comparisons of the numbers of DEGs at various time points. **Table S4.** GO term enrichment of DEGs between various time points. **Table S5.** Five hub genes in the blue module. **Table S6.** Seven hub genes in the turquoise module. **Table S7.** GO term enrichment of the hub genes in the blue module. **Table S8.** KEGG pathway enrichment of the hub genes in the blue module. **Table S9.** KEGG pathway classification of the hub genes in the blue module. **Table S10.** Prediction of the transcription factors in the blue module. **Table S11.** GO term enrichment of the hub genes in the turquoise module. **Table S12.** KEGG pathway enrichment of the hub genes in the turquoise module. **Table S13.** KEGG pathway classification of the hub genes in the turquoise module. **Table S14.** Prediction of the transcription factors in the turquoise module. **Table S15.** Gene expression changes of secondary metabolites in DEGs at different time period.

## Data Availability

All analysis tools used in the study are publicly available. The original data were deposited in the NCBI database (https://www.ncbi.nlm.nih.gov/bioproject/) and the accession is PRJNA549113.
