# Supplementary material for: Transcriptomics and co-expression network analysis revealing candidate genes for the laccase activity of Trametes gibbosa
Source: BMC Microbiol. 2023 Jan 26;23:29. doi: 10.1186/s12866-022-02727-3 (PMC9878871; doi:10.1186/s12866-022-02727-3)
Supplement: Supplementary file 1 — Additional file 1: Fig. S1. Principal components analysis of variation and correlation coefficient analysis. Fig. S2. The upset plot shows the enrichment in DEGs in each group (P_value<0.05). Set Size represents the number of go terms. Fig. S3. Quantitative real-time polymerase chain reaction. A. The gene expression of qRT-PCR and RNA_seq. The short line represents the standard deviations on the column in qRT-PCR. B. Gene expression correlation between RNA-Seq and qRT-PCR data. Each blue dot indicates the selected genes. Fig. S4. Clustering of module eigengenes. Fig. S5. Modules significance correlation. Fig. S6. Interaction of co-expression patterns of the genes in the blue module. Fig. S7. Selection of the soft-thresholding power. A. The panel shows the scale-free fit index versus soft-thresholding power. B. The left panel displays the mean connectivity versus soft-thresholding power. C. Verification of the memory network using the selected values. R2=0.85. Fig. S8. Clustering dendrograms of detected genes and modules. Note: different colors represent different modules. Table S1. Primers used in the present study. Table S2. Transcriptome sequencing data statistics. Table S3. Comparisons of the numbers of DEGs at various time points. Table S4. GO term enrichment of DEGs between various time points. Table S5. Five hub genes in the blue module. Table S6. Seven hub genes in the turquoise module. Table S7. GO term enrichment of the hub genes in the blue module. Table S8. KEGG pathway enrichment of the hub genes in the blue module. Table S9. KEGG pathway classification of the hub genes in the blue module. Table S10. Prediction of the transcription factors in the blue module. Table S11. GO term enrichment of the hub genes in the turquoise module. Table S12. KEGG pathway enrichment of the hub genes in the turquoise module. Table S13. KEGG pathway classification of the hub genes in the turquoise module. Table S14. Prediction of the transcription factors in the turquoise mo [file 12866_2022_2727_MOESM1_ESM.pdf]

Supplemental materials legends

Fig. S1. Principal components analysis of variation and correlation coefficient analysis;

Fig. S2. The upset plot shows the enrichment in DEGs in each group (P_value<0.05). Set Size represents the number of go terms.

Fig. S3. Quantitative real-time polymerase chain reaction. A. The gene expression of qRT-PCR and RNA_seq. The short line represents the standard deviations on the column in qRT-PCR. B. Gene expression correlation between RNA-Seq and qRT-PCR data. Each blue dot indicates the selected genes.

Fig. S4. Clustering of module eigengenes;

Fig. S5. Modules significance correlation;

Fig. S6. Interaction of co-expression patterns of the genes in the blue module;

Fig. S7. Selection of the soft-thresholding power. A. The panel shows the scale-free fit index versus soft-thresholding power; B. The left panel displays the mean connectivity versus soft-thresholding power; C. Verification of the memory network using the selected values; R^2=0.85.

Fig. S8. Clustering dendrograms of detected genes and modules. Note: different colors represent different modules

Table S1. Primers used in the present study

Table S2. Transcriptome sequencing data statistics;

Table S3. Comparisons of the numbers of DEGs at various time points;

Table S4. GO term enrichment of DEGs between various time points;

Table S5. Five hub genes in the blue module;

Table S6. Seven hub genes in the turquoise module;

Table S7. GO term enrichment of the hub genes in the blue module;

Table S8. KEGG pathway enrichment of the hub genes in the blue module;

Table S9. KEGG pathway classification of the hub genes in the blue module;

Table S10. Prediction of the transcription factors in the blue module;

Table S11. GO term enrichment of the hub genes in the turquoise module;

Table S12. KEGG pathway enrichment of the hub genes in the turquoise module;

Table S13. KEGG pathway classification of the hub genes in the turquoise module;

Table S14. Prediction of the transcription factors in the turquoise module;

Table S15. Gene expression changes of secondary metabolites in DEGs at different time period.

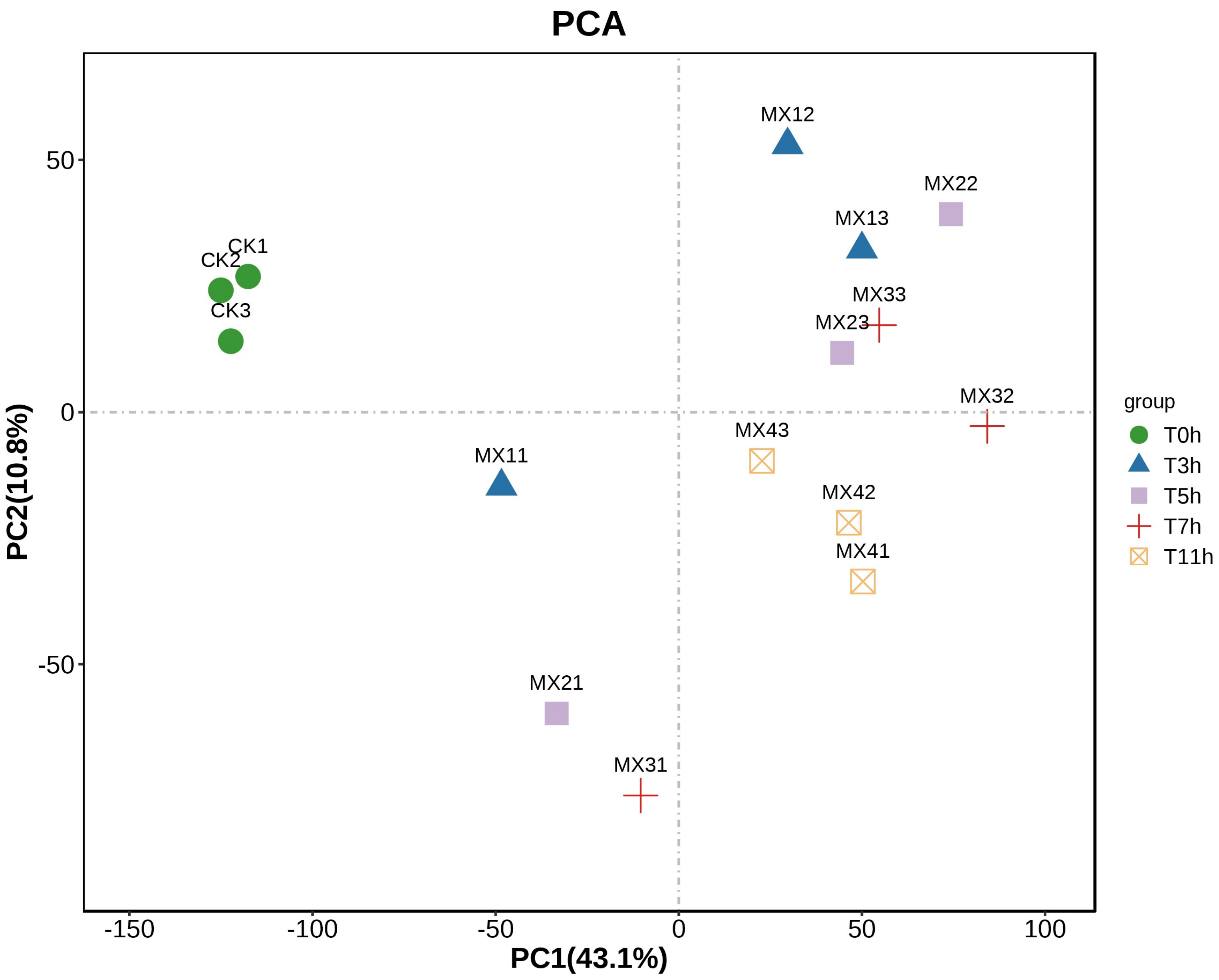

Fig. S1. Principal components analysis of variation and correlation coefficient analysis

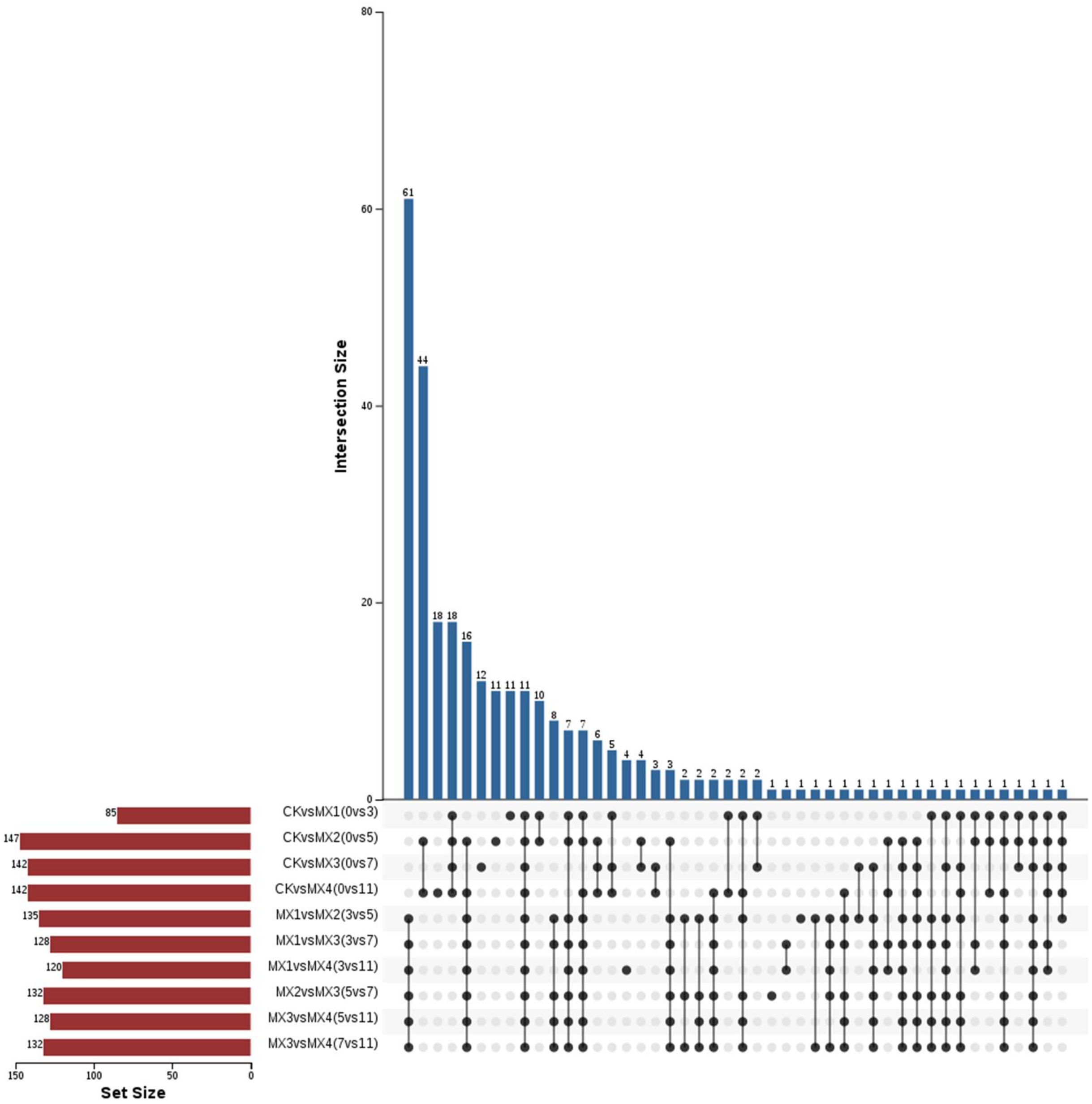

Fig. S2. The upset plot shows the enrichment in DEGs in each group (P_value<0.05). Set Size represents the number of go terms.

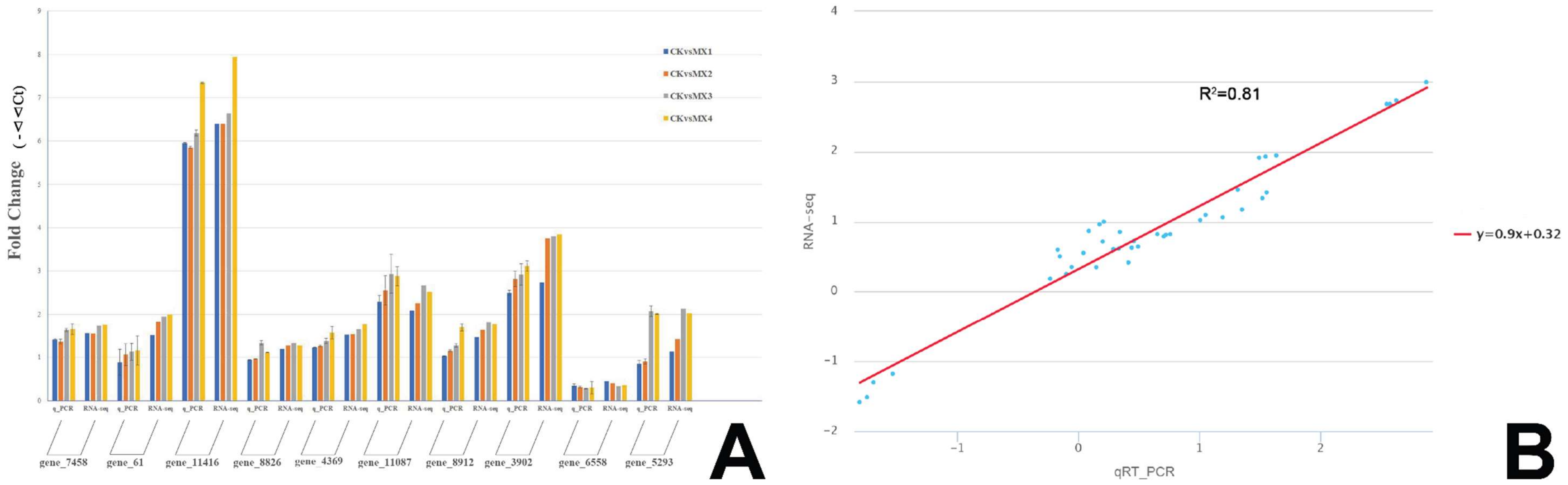

Fig. S3. Quantitative real-time polymerase chain reaction. A. The gene expression of qRT-PCR and RNA_seq. The short line represents the standard deviations on the column in qRT-PCR. B. Gene expression correlation between RNA-Seq and qRT-PCR data. Each blue dot indicates the selected genes.

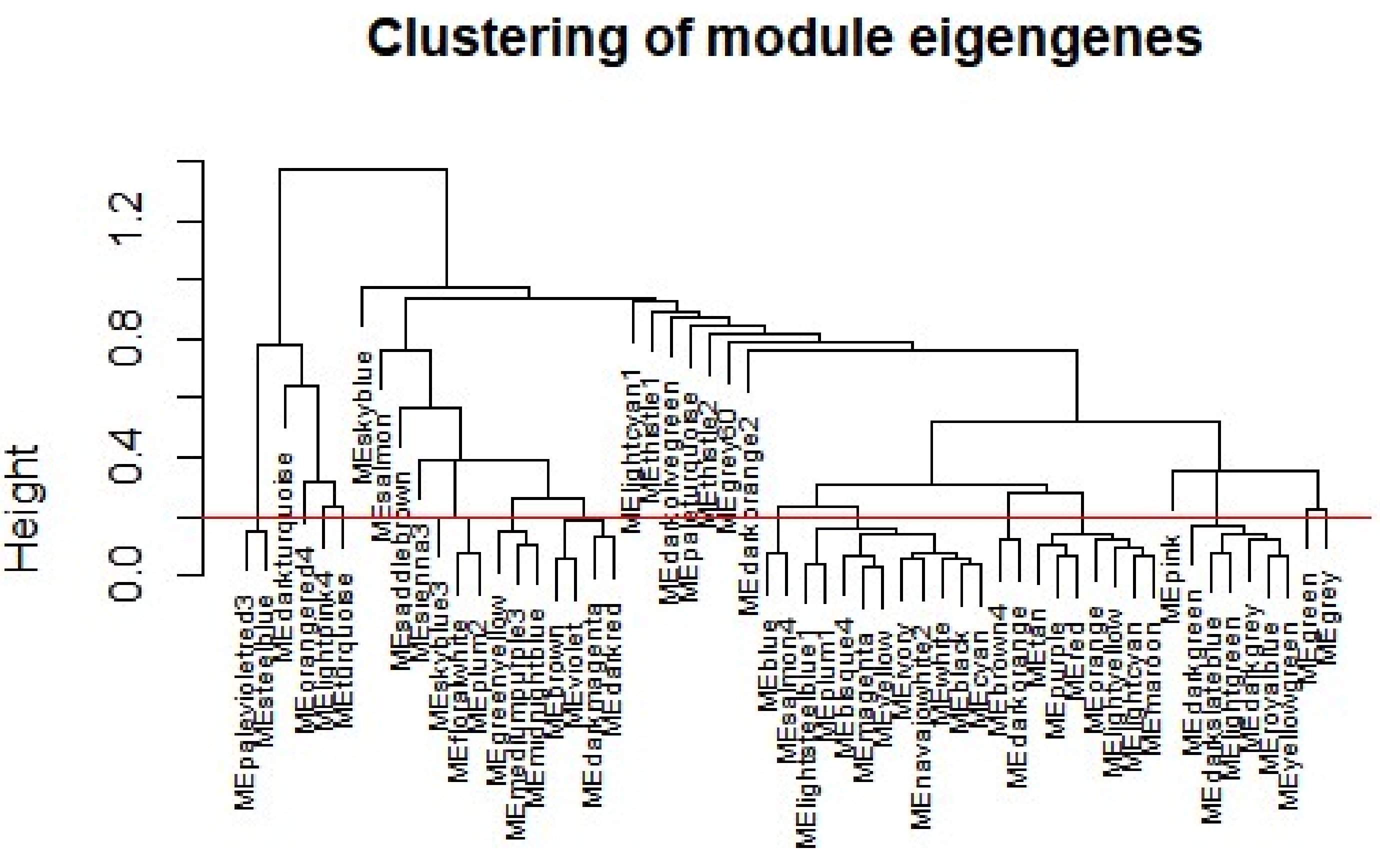

Fig. S4. Clustering of module eigengenes

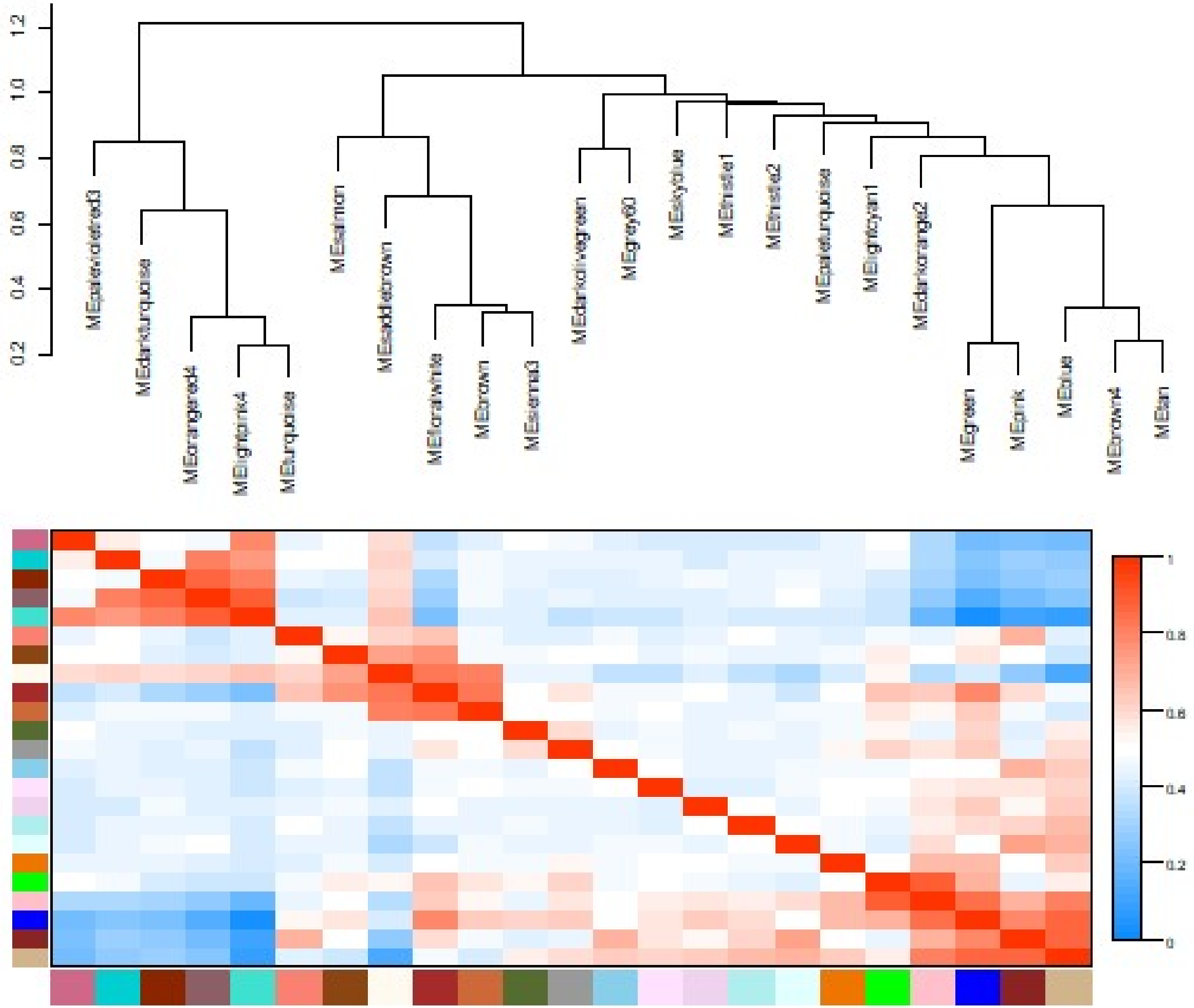

Fig. S5. Modules significance correlation

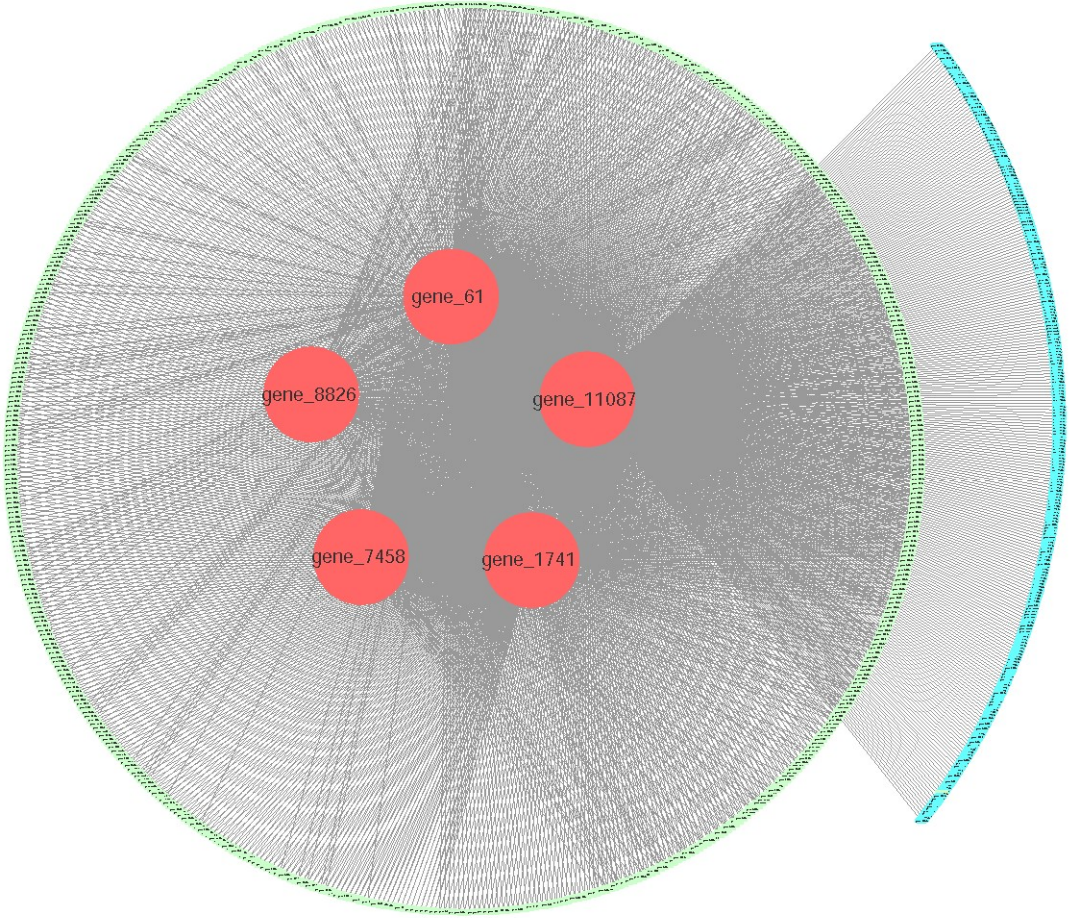

Fig. S6. Interaction of co-expression patterns of the genes in the blue module:

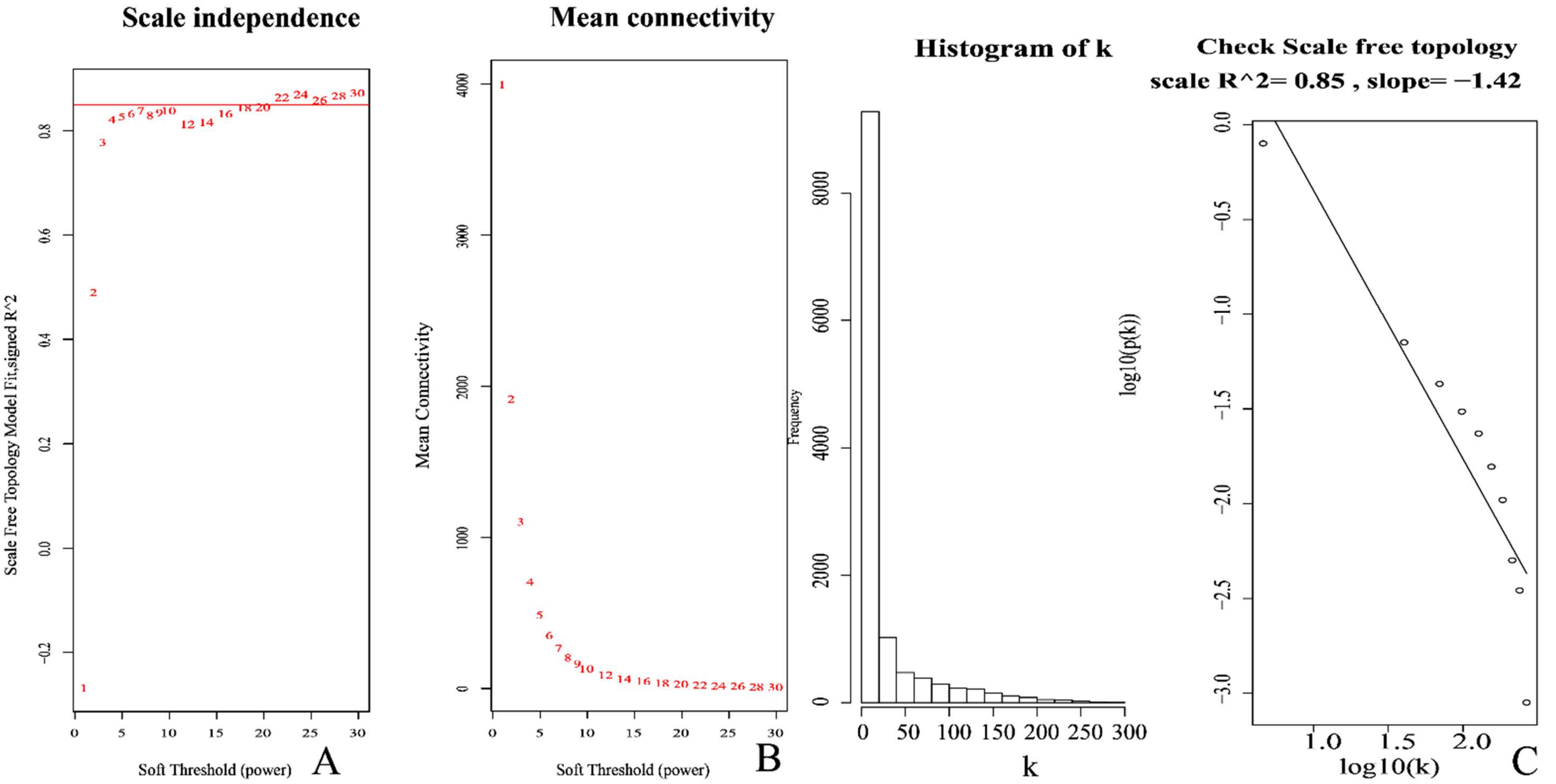

Fig.S7. Selection of the soft-thresholding power. A. The panel shows the scale-free fit index versus soft-thresholding power; B. The left panel displays the mean connectivity versus soft-thresholding power; C. Verification of the memory network using the selected values; R^2=0.85.

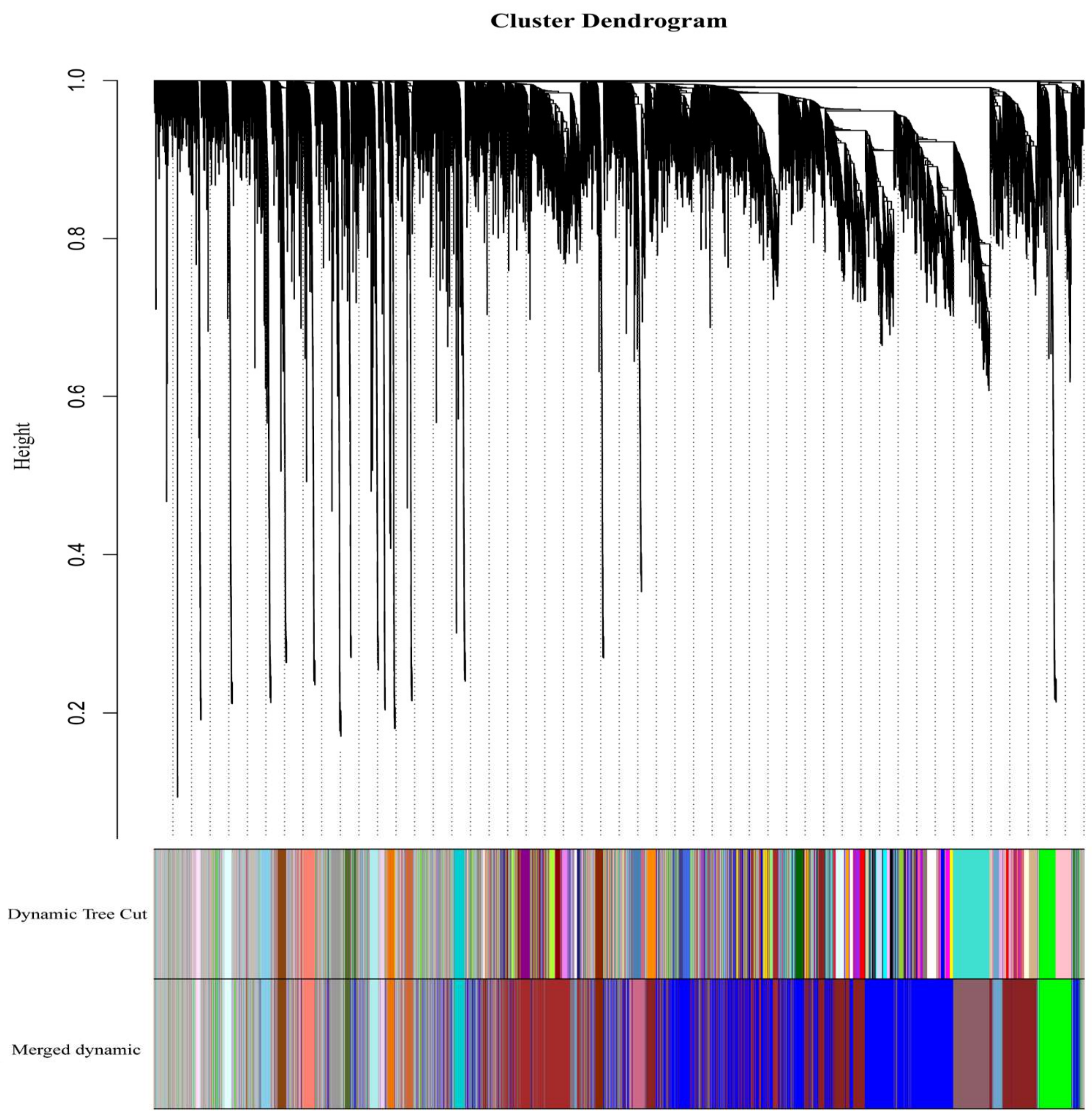

Fig.S8. Clustering dendrograms of detected genes and modules. Note: different colors represent different module

Table S1. Primers used in the present study

Gene name	Gene ID	Primers Sequences (5′-3′)
gene_11087	jgi.p|Tragib1|1400843	F: 5' CTTGAGGTATGACGGTCAGATG 3'<br>R: 5' GGCGAAGTTCCACGATAGGT 3'
gene_61	jgi.p|Tragib1|1414099	F: 5' CCATTCCATCGCTTCCTACG 3'<br>R: 5'GATTGTAGAGTGCCGTGAAGG 3'
gene_8826	jgi.p|Tragib1|1387922	F: 5' CAGGAGCAGAAGGAATGGATG 3'<br>R: 5'AACAGAGCCTCGGAGAACTT 3'
gene_7458	jgi.p|Tragib1|1323325	F: 5' CTGCTCTTCCTGGTCCTCAA 3'<br>R: 5' GCTCTCCACATTGCCATCTT 3'
gene_4369	jgi.p|Tragib1|1316018	F: 5' TCCTCCCTTAGAACCACTG 3'<br>R: 5'GCCGATACTTGTCCGAAT 3'
gene_8912	jgi.p|Tragib1|1326595	F: 5' CTTGATGAACGAGTTGACCAGA 3'<br>R: 5'CCGAGCGTGTAGTCCATTC 3'
gene_5293	jgi.p|Tragib1|1318729	F: 5' CTCTGTATTCGTGGCGTTTG 3'<br>R: 5' ATCACCATAGTTGCCGTCTGT 3
gene_3092	jgi.p|Tragib1|1003737	F: 5' CGGTCCTTCTCCAGATTCTCA 3'<br>R: 5' TTGTCGTAGTTGTACTCGGTGC 3'
gene_6558	*jgi.p|Tragib1|1419565*	F: 5'TCGTAAGATACCCATTCGC 3'<br>R: 5'CGTAGGGATAACTGGAGACAA 3'
gene_11416	*jgi.p|Tragib1|1413701*	F: 5'ACCAGTGCGTAAGACAGCCAAT 3'<br>R: 5'CGTCCAGTTGCGATGACCTTGT 3'
Gpd		F: 5'AACGGTTTCGGTCGTATCGG 3'<br>R: 5'CTTGCCCTCGACCCAGAGCT 3'

Table S2. Transcriptome sequencing data statistics

sample	Total clean bases	Total clean reads	GC percentage(%)	Q30 percentage(%)	Mapped Reads
CK1	6,688,035,648	22,373,688	59.98%	92.74%	39,666,505 (88.65%)
CK2	6,161,676,562	20,654,264	60.22%	93.16%	36,900,678 (89.33%)
CK3	6,511,639,200	21,775,420	60.29%	93.12%	38,969,090 (89.48%)
MX11	6,323,330,772	21,160,449	60.08%	93.00%	37,458,653 (88.51%)
MX12	8,347,223,964	27,906,550	60.14%	92.34%	50,163,296 (89.88%)
MX13	8,709,331,142	23,090,636	60.14%	92.87%	53,159,264 (91.37%)
MX21	6,519,256,502	32,833,128	60.03%	92.54%	38,894,913 (89.07%)
MX22	9,205,380,236	30,773,780	60.13%	92.07%	55,420,730 (90.05%)
MX23	8,584,493,006	28,691,572	59.68%	92.29%	52,042,156 (90.69%)
MX31	6,743,734,040	22,562,342	59.75%	92.68%	40,277,507 (89.26%)
MX32	8,721,228,424	29,134,517	60.11%	91.84%	52,740,620 (90.51%)
MX33	8,358,662,628	23,939,760	60.21%	92.12%	50,379,387 (90.16%)
MX41	7,710,177,650	25,764,611	60.15%	92.29%	46,242,536 (89.74%)
MX42	8,300,000,096	27,749,266	60.12%	92.09%	49,690,309 (89.53%)
MX43	7,667,128,650	26,633,479	60.05%	92.29%	46,011,454 (89.75%)

Mapped Reads: the number of Reads which mapped to the reference genome, including a unique location and multiple locations in the reference genome, and percentage of Clean Reads.

Table S3. Comparisons of the numbers of DEGs at various time points

DEG set	All DEG	Up-regulated	Down-regulated
Group CK_vs_Group MX1	1124	367	757
Group CK_vs_Group MX2	1084	345	739
Group CK_vs_Group MX3	1356	466	890
Group CK_vs_Group MX4	1311	460	851
Group MX1_vs_Group MX2	27	13	14
Group MX1_vs_Group MX3	65	19	46
Group MX1_vs_Group MX4	152	68	84
Group MX2_vs_Group MX3	21	9	12
Group MX2_vs_Group MX4	43	22	21
Group MX3_vs_Group MX4	49	34	15
Total	5232	1803	3429

Table S4. GO term enrichment of DEGs between various time points

Catgory	GO_classify2	GO_ID	CKvs MX1	CKvs MX2	CKvs MX3	CKvs MX4	MX1vs MX2	MX1vs MX3	MX1vs MX4	MX2vs MX3	MX2vs MX4	MX3vs MX4
BP	cellular process	GO:0009987	197	195	266	15	3	9	15	4	6	5
BP	metabolic process	GO:0008152	261	253	335	22	3	11	22	2	6	6
BP	single-organism process	GO:0044699	182	181	233	18	3	11	18	1	4	5
BP	localization	GO:0051179	77	74	87	11	3	6	11	1	1	2
BP	biological regulation	GO:0065007	73	66	87	9	1	5	9	1	1	3
BP	response to stimulus	GO:0050896	54	49	70	4	1	3	4	1	1	2
BP	signaling	GO:0023052	12	9	12	2	1	2	2	1	1	2
BP	cellular component organization or biogenesis	GO:0071840	43	48	70	1	0	1	1	0	0	0
BP	reproduction	GO:0000003	9	7	12	1	0	1	1	0	0	0
BP	reproductive process	GO:0022414	9	7	12	1	0	1	1	0	0	0
BP	detoxification	GO:0098754	7	8	11	1	0	1	1	0	0	0
BP	multi-organism process	GO:0051704	3	3	2	1	0	0	1	0	0	0
BP	developmental process	GO:0032502	2	3	3	0	0	0	0	0	0	0
CC	cell	GO:0005623	158	166	229	11	2	6	11	2	6	5
CC	cell part	GO:0044464	157	166	228	11	2	6	11	2	7	5
CC	membrane	GO:0016020	156	161	192	27	3	10	27	2	4	6
CC	membrane part	GO:0044425	111	123	144	17	2	9	17	1	2	4
CC	organelle	GO:0043226	108	116	166	5	1	2	5	1	3	1
CC	organelle part	GO:0044422	55	64	103	2	1	2	2	0	2	1
CC	macromolecular complex	GO:0032991	43	52	75	1	0	0	1	1	1	1
CC	membrane-enclosed lumen	GO:0031974	20	22	32	0	0	0	0	0	0	0
CC	extracellular region	GO:0005576	7	6	6	3	0	0	3	0	0	0
CC	supramolecular complex	GO:0099080	3	4	4	0	0	0	0	0	0	0
CC	extracellular region part	GO:0044421	1	1	1	0	0	0	0	0	0	0
CC	nucleoid	GO:0009295	1	2	3	0	0	0	0	0	0	0
MF	catalytic activity	GO:0003824	287	270	338	27	3	12	27	0	5	8
MF	binding	GO:0005488	230	225	294	20	6	10	20	3	8	7
MF	transporter activity	GO:0005215	31	34	33	3	0	1	3	0	0	0
MF	structural molecule activity	GO:0005198	9	12	11	2	0	0	2	1	4	2

MF	nucleic acid binding transcription factor activity	GO:0001071	8	6	7	1	0	1	1	0	0	0
MF	molecular function regulator	GO:0098772	7	3	5	0	0	0	0	0	0	0
MF	antioxidant activity	GO:0016209	6	7	10	1	0	1	1	0	0	0
MF	signal transducer activity	GO:0004871	5	4	4	0	0	0	0	0	0	1
MF	transcription factor activity, protein binding	GO:0000988	2	2	2	0	0	0	0	0	0	0
MF	electron carrier activity	GO:0009055	2	4	5	0	0	0	0	0	0	0
MF	molecular transducer activity	GO:0060089	1	0	0	0	0	0	0	0	0	0
MF	nutrient reservoir activity	GO:0045735	1	0	1	0	0	0	0	0	0	0
MF	protein tag	GO:0031386	1	1	1	0	0	0	0	0	0	0

Table S5. Five hub genes in the blue module

Gene ID	MM.blue	GS.	K.in	moduleColors
gene_8826	0.97653	0.951119	123.0598	blue
gene_7458	0.962732	0.928418	116.7773	blue
gene_61	0.970936	0.98198	96.75531	blue
gene_1741	-0.82067	0.921505	96.47252	blue
gene_11087	0.968214	0.921994	130.5777	blue

Table S6. Seven hub genes in the turquoise module;

Gene ID	MM.turquoise	GS.	K.in	module Colors
gene_61	-0.9155342	0.98198	96.75531308	turquoise
gene_8826	-0.945376795	0.951119	123.0597578	turquoise
gene_4369	-0.917144557	0.949823	99.14629039	turquoise
gene_8912	-0.946864288	0.9466	100.5721476	turquoise
gene_5464	-0.935925875	0.94006	89.94963133	turquoise
gene_9218	-0.877565284	0.938749	125.4794949	turquoise
gene_7458	-0.914459977	0.928418	116.7772787	turquoise

Table S7. GO term enrichment of the hub genes in the blue module;

Catogry	GO.ID	Term	Annotated	Significant	Expected	KS
BP	GO:0042026	protein refolding	5	5	0.72	0.00032
BP	GO:0030433	ER-associated ubiquitin-dependent protein catabolic process	11	6	1.59	0.00132
BP	GO:0022402	cell cycle process	161	47	23.27	0.00147
BP	GO:0016125	sterol metabolic process	13	4	1.88	0.00174
BP	GO:0071310	cellular response to organic substance	21	8	3.03	0.00215
BP	GO:0000710	meiotic mismatch repair	4	4	0.58	0.00215
BP	GO:0006302	double-strand break repair	35	14	5.06	0.00279
BP	GO:0007020	microtubule nucleation	6	4	0.87	0.00383
BP	GO:0016043	cellular component organization	571	129	82.52	0.00432
BP	GO:0006352	DNA-templated transcription, initiation	31	12	4.48	0.00449
BP	GO:0009628	response to abiotic stimulus	42	16	6.07	0.00494
CC	GO:0044428	nuclear part	391	107	59.55	0.00024
CC	GO:0005741	mitochondrial outer membrane	28	5	4.26	0.00104
CC	GO:0035861	site of double-strand break	8	6	1.22	0.00148
CC	GO:0031981	nuclear lumen	284	71	43.26	0.00172
CC	GO:0005694	chromosome	162	54	24.67	0.00213
CC	GO:0043234	protein complex	633	131	96.41	0.00251
CC	GO:0043231	intracellular membrane-bounded organelle	1479	279	225.27	0.00409
CC	GO:0000781	chromosome, telomeric region	24	11	3.66	0.00467
MF	GO:0003723	RNA binding	246	47	31.77	5.60E-05
MF	GO:0005524	ATP binding	441	95	56.95	0.00044
MF	GO:0000104	succinate dehydrogenase activity	4	4	0.52	0.00165
MF	GO:0004842	ubiquitin-protein transferase activity	18	6	2.32	0.00539
MF	GO:0051082	unfolded protein binding	37	12	4.78	0.00597
MF	GO:0043566	structure-specific DNA binding	32	15	4.13	0.00656
MF	GO:0005515	protein binding	259	49	33.45	0.00691
MF	GO:0016639	oxidoreductase activity, acting on the CH-NH2 group of donors, NAD or NADP as acceptor	3	3	0.39	0.00769

MF	GO:0000175	3'-5'-exoribonuclease activity	5	2	0.65	0.00863
MF	GO:0003682	chromatin binding	31	11	4	0.00864
MF	GO:0004521	endoribonuclease activity	20	3	2.58	0.00866
MF	GO:0000400	four-way junction DNA binding	3	3	0.39	0.00868
MF	GO:0003873	6-phosphofructo-2-kinase activity	3	3	0.39	0.00914
MF	GO:0005509	calcium ion binding	35	7	4.52	0.00919
MF	GO:0031072	heat shock protein binding	5	4	0.65	0.00931
MF	GO:0000166	nucleotide binding	834	150	107.71	0.00951
MF	GO:0009378	four-way junction helicase activity	5	4	0.65	0.00964
MF	GO:0016892	endoribonuclease activity, producing 3'-phosphomonoesters	5	2	0.65	0.01579
MF	GO:0016894	endonuclease activity, active with either ribo- or deoxyribonucleic acids and producing 3'-phosphomonoesters	5	2	0.65	0.01579
MF	GO:0017048	Rho GTPase binding	3	2	0.39	0.01948
MF	GO:0017016	Ras GTPase binding	15	4	1.94	0.02037
MF	GO:0031267	small GTPase binding	15	4	1.94	0.02037
MF	GO:0051087	chaperone binding	9	5	1.16	0.02075
MF	GO:0003697	single-stranded DNA binding	18	8	2.32	0.02175
MF	GO:0004722	protein serine/threonine phosphatase activity	10	4	1.29	0.02196
MF	GO:0043167	ion binding	1301	209	168.02	0.02234
MF	GO:0008177	succinate dehydrogenase (ubiquinone) activity	2	2	0.26	0.02253
MF	GO:0005488	binding	2170	329	280.25	0.02726
MF	GO:0030515	snoRNA binding	12	6	1.55	0.03057
MF	GO:0000403	Y-form DNA binding	2	2	0.26	0.03279
MF	GO:0004180	carboxypeptidase activity	25	4	3.23	0.03343
MF	GO:0003958	NADPH-hemoprotein reductase activity	5	1	0.65	0.03392
MF	GO:0033897	ribonuclease T2 activity	2	2	0.26	0.0349
MF	GO:0051787	misfolded protein binding	2	2	0.26	0.03543
MF	GO:0008238	exopeptidase activity	47	5	6.07	0.03743
MF	GO:0004352	glutamate dehydrogenase (NAD+) activity	2	2	0.26	0.03898
MF	GO:0050660	flavin adenine dinucleotide binding	62	11	8.01	0.03936
MF	GO:0003690	double-stranded DNA binding	13	6	1.68	0.04045

MF	GO:0033677	DNA/RNA helicase activity	4	3	0.52	0.04128
MF	GO:0033679	3'-5' DNA/RNA helicase activity	4	3	0.52	0.04128
MF	GO:0000990	core RNA polymerase binding transcription factor activity	4	3	0.52	0.04141
MF	GO:0000991	core RNA polymerase II binding transcription factor activity	4	3	0.52	0.04141
MF	GO:0016635	oxidoreductase activity, acting on the CH-CH group of donors, quinone or related compound as acceptor	3	2	0.39	0.04165
MF	GO:0032135	DNA insertion or deletion binding	2	2	0.26	0.04227
MF	GO:0032137	guanine/thymine mispair binding	2	2	0.26	0.04227
MF	GO:0032138	single base insertion or deletion binding	2	2	0.26	0.04227
MF	GO:0008536	Ran GTPase binding	11	2	1.42	0.04294
MF	GO:0008135	translation factor activity, nucleic acid binding	60	8	7.75	0.04501
MF	GO:0047750	cholestenol delta-isomerase activity	2	2	0.26	0.0453
MF	GO:0051020	GTPase binding	16	4	2.07	0.046
MF	GO:0019888	protein phosphatase regulator activity	2	2	0.26	0.04705
MF	GO:0060090	binding, bridging	8	4	1.03	0.04802
MF	GO:0030674	protein binding, bridging	8	4	1.03	0.04802
MF	GO:0019899	enzyme binding	32	9	4.13	0.04952

Table S8. KEGG pathway enrichment of the hub genes in the blue module

Kegg_pathway	ko_id	P-value	rich_factor
Cell cycle - yeast	ko04111	4.09E-06	2.348965575
Meiosis - yeast	ko04113	0.000208476	2.305521697
Citrate cycle (TCA cycle)	ko00020	0.000839183	2.768423714
Non-homologous end-joining	ko03450	0.002326015	3.706484642
DNA replication	ko03030	0.002565169	2.265073948
Protein processing in endoplasmic reticulum	ko04141	0.004240737	1.783745734
Mismatch repair	ko03430	0.00489029	2.548208191
mRNA surveillance pathway	ko03015	0.012151695	1.78235327
Nicotinate and nicotinamide metabolism	ko00760	0.032760599	2.162116041
Sulfur relay system	ko04122	0.064928388	2.470989761
Spliceosome	ko03040	0.07290363	1.386779968
Basal transcription factors	ko03022	0.073538764	1.753605637
Regulation of autophagy	ko04140	0.112280966	1.887561623
Nucleotide excision repair	ko03420	0.137055315	1.491634063
Glycosylphosphatidylinositol(GPI)-anchor biosynthesis	ko00563	0.159533701	1.698805461
Ubiquitin mediated proteolysis	ko04120	0.160750224	1.359044369
Nitrogen metabolism	ko00910	0.167965482	1.812059158
Ribosome biogenesis in eukaryotes	ko03008	0.17501252	1.30319323
Arginine biosynthesis	ko00220	0.199587503	1.698805461
Homologous recombination	ko03440	0.213381469	1.544368601
Taurine and hypotaurine metabolism	ko00430	0.216713154	2.265073948
Sesquiterpenoid and triterpenoid biosynthesis	ko00909	0.272730806	3.397610922
Tyrosine metabolism	ko00350	0.302556117	1.359044369
RNA transport	ko03013	0.303622696	1.143121432
Inositol phosphate metabolism	ko00562	0.374156528	1.29432797
Lipoic acid metabolism	ko00785	0.379864981	2.265073948
Regulation of mitophagy - yeast	ko04139	0.384097692	1.199156796
ABC transporters	ko02010	0.390829589	1.510049298
Aminoacyl-tRNA biosynthesis	ko00970	0.412287373	1.164895173
Pyrimidine metabolism	ko00240	0.424999616	1.087235495
2-Oxocarboxylic acid metabolism	ko01210	0.440349749	1.132536974
Base excision repair	ko03410	0.445181402	1.181777712
Carbon metabolism	ko01200	0.465931463	1.045418745
Proteasome	ko03050	0.495581151	1.072929765
Steroid biosynthesis	ko00100	0.507218588	1.132536974
Phosphatidylinositol signaling system	ko04070	0.507218588	1.132536974
RNA polymerase	ko03020	0.518456788	1.061753413
Folate biosynthesis	ko00790	0.546015305	1.132536974
Lysine degradation	ko00310	0.546302427	1.045418745
Glycerophospholipid metabolism	ko00564	0.548882187	1.019283276
alpha-Linolenic acid metabolism	ko00592	0.549229883	1.359044369
Arachidonic acid metabolism	ko00590	0.549229883	1.359044369
C5-Branched dibasic acid metabolism	ko00660	0.549229883	1.359044369
RNA degradation	ko03018	0.574201531	0.988395904
Pantothenate and CoA biosynthesis	ko00770	0.582187312	1.019283276
One carbon pool by folate	ko00670	0.591281429	1.045418745
Cyanoamino acid metabolism	ko00460	0.591281429	1.045418745

Glycolysis / Gluconeogenesis	ko00010	0.59953149	0.970745978
Fructose and mannose metabolism	ko00051	0.616859109	0.970745978
Selenocompound metabolism	ko00450	0.672453714	0.970745978
Alanine, aspartate and glutamate metabolism	ko00250	0.680193029	0.886333284
Peroxisome	ko04146	0.683109185	0.89748213
Fatty acid biosynthesis	ko00061	0.720826225	0.84940273
Starch and sucrose metabolism	ko00500	0.721374353	0.864846416
Ether lipid metabolism	ko00565	0.762075804	0.755024649
Ubiquinone and other terpenoid-quinone biosynthesis	ko00130	0.762075804	0.755024649
Glutathione metabolism	ko00480	0.763007088	0.808954981
Porphyrin and chlorophyll metabolism	ko00860	0.767443044	0.755024649
SNARE interactions in vesicular transport	ko04130	0.793542298	0.71528651
Ascorbate and aldarate metabolism	ko00053	0.797248251	0.679522184
Biotin metabolism	ko00780	0.797248251	0.679522184
Glyoxylate and dicarboxylate metabolism	ko00630	0.823115289	0.702953984
Riboflavin metabolism	ko00740	0.827236268	0.61774744
Biosynthesis of antibiotics	ko01130	0.837357414	0.864846416
MAPK signaling pathway - yeast	ko04011	0.84332488	0.765658799
Oxidative phosphorylation	ko00190	0.845125234	0.774139197
Biosynthesis of amino acids	ko01230	0.847015273	0.803071672
Biosynthesis of unsaturated fatty acids	ko01040	0.873786309	0.590888856
Lysine biosynthesis	ko00300	0.874595176	0.522709373
Purine metabolism	ko00230	0.875600308	0.755024649
Glycerolipid metabolism	ko00561	0.902117293	0.543617747
Butanoate metabolism	ko00650	0.909003795	0.45301479
Pyruvate metabolism	ko00620	0.917654561	0.566268487
Propanoate metabolism	ko00640	0.922496653	0.424701365
Valine, leucine and isoleucine biosynthesis	ko00290	0.922496653	0.424701365
beta-Alanine metabolism	ko00410	0.922496653	0.424701365
Amino sugar and nucleotide sugar metabolism	ko00520	0.925298268	0.61774744
Sulfur metabolism	ko00920	0.933994618	0.399718932
Various types of N-glycan biosynthesis	ko00513	0.933994618	0.399718932
Fatty acid degradation	ko00071	0.94190205	0.468635989
N-Glycan biosynthesis	ko00510	0.94190205	0.468635989
Galactose metabolism	ko00052	0.94379177	0.377512325
Valine, leucine and isoleucine degradation	ko00280	0.949128374	0.45301479
Histidine metabolism	ko00340	0.952138962	0.357643255
Phenylalanine, tyrosine and tryptophan biosynthesis	ko00400	0.952138962	0.357643255
Phenylalanine metabolism	ko00360	0.952138962	0.357643255
Cysteine and methionine metabolism	ko00270	0.954029122	0.497211354
Sphingolipid metabolism	ko00600	0.959250161	0.339761092
Fatty acid metabolism	ko01212	0.96109627	0.424701365
Protein export	ko03060	0.970467646	0.30887372
Arginine and proline metabolism	ko00330	0.974133905	0.388298391
Methane metabolism	ko00680	0.978604813	0.283134243
Pentose and glucuronate interconversions	ko00040	0.981791793	0.271808874
Tryptophan metabolism	ko00380	0.987074517	0.339761092

Endocytosis	ko04144	0.988780663	0.465426154
Phagosome	ko04145	0.99024694	0.323581993
Glycine, serine and threonine metabolism	ko00260	0.996929792	0.188756162
Ribosome	ko03010	0.999997602	0.182014871

Table S9. KEGG pathway classification of the hub genes in the blue module

KEGG Category	ko.ID	KEGG Category2	gene Count
Cellular Processes 44 (42)	ko04111	Cell cycle - yeast	28
	ko04113	Meiosis - yeast	19
	ko04144	Endocytosis	5
	ko04146	Peroxisome	7
	ko04145	Phagosome	2
Environmental Information Processing 13 (13)	ko02010	ABC transporters	2
	ko04011	MAPK signaling pathway-yeast	8
	ko04070	Phosphatidylinositol signaling system	3
Genetic Information Processing 152 (146)	ko03050	Proteasome	6
	ko03060	Protein export	1
	ko04141	Protein processing in endoplasmic reticulum	21
	ko03018	RNA degradation	8
	ko04130	SNARE interactions in vesicular transport	2
	ko04122	Sulfur relay system	4
	ko04120	Ubiquitin mediated proteolysis	12
	ko03410	Base excision repair	4
	ko03030	DNA replication1	13
	ko03440	Homologous recombination	5
	ko03430	Mismatch repair	9
	ko03450	Non-homologous end-joining	6
	ko03420	Nucleotide excision repair	9
	ko03022	Basal transcription factors	8
	ko03020	RNA polymerase	5
	ko03040	Spliceosome	20
	ko00970	Aminoacyl-tRNA biosynthesis	6
	ko03013	RNA transport1	18
	ko03010	Ribosome	3
	ko03008	Ribosome biogenesis in eukaryotes	14
	ko03015	mRNA surveillance pathway	16
Metabolism 101 (93)	ko00250	Alanine, aspartate and glutamate metabolism	3
	ko00330	Arginine and proline metabolism	2
	ko00220	Arginine biosynthesis	4
	ko00270	Cysteine and methionine metabolism	3
	ko00260	Glycine, serine and threonine metabolism	1
	ko00340	Histidine metabolism	1
	ko00300	Lysine biosynthesis	1
	ko00310	Lysine degradation	4
	ko00360	Phenylalanine metabolism	1
	ko00400	Phenylalanine, tyrosine and tryptophan biosynthesis	1
	ko00380	Tryptophan metabolism	2
	ko00350	Tyrosine metabolism	5
	ko00290	Valine, leucine and isoleucine biosynthesis	1
	ko00280	Valine, leucine and isoleucine degradation	2
	ko00520	Amino sugar and nucleotide sugar metabolism	5
	ko00053	Ascorbate and aldarate metabolism	1
	ko00650	Butanoate metabolism	1
	ko00660	C5-Branched dibasic acid metabolism	1
	ko00020	Citrate cycle (TCA cycle)	11
	ko00051	Fructose and mannose metabolism	3
	ko00052	Galactose metabolism	1
	ko00010	Glycolysis / Gluconeogenesis	6
	ko00630	Glyoxylate and dicarboxylate metabolism	3

ko00562	Inositol phosphate metabolism	4
ko00040	Pentose and glucuronate interconversions	1
ko00640	Propanoate metabolism	1
ko00620	Pyruvate metabolism	3
ko00500	Starch and sucrose metabolism	7
ko00680	Methane metabolism	1
ko00910	Nitrogen metabolism	4
ko00190	Oxidative phosphorylation	9
ko00920	Sulfur metabolism	1
ko01210	2-Oxocarboxylic acid metabolism	6
ko01230	Biosynthesis of amino acids	13
ko01130	Biosynthesis of antibiotics	28
ko01200	Carbon metabolism	16
ko01212	Fatty acid metabolism	2
ko00510	N-Glycan biosynthesis	2
ko00513	Various types of N-glycan biosynthesis	1
ko00590	Arachidonic acid metabolism	1
ko01040	Biosynthesis of unsaturated fatty acids	2
ko00565	Ether lipid metabolism	1
ko00061	Fatty acid biosynthesis	1
ko00071	Fatty acid degradation	2
ko00561	Glycerolipid metabolism	2
ko00564	Glycerophospholipid metabolism	6
ko00600	Sphingolipid metabolism	1
ko00100	Steroid biosynthesis	3
ko00592	alpha-Linolenic acid metabolism	1
ko00780	Biotin metabolism	1
ko00790	Folate biosynthesis	2
ko00785	Lipoic acid metabolism	1
ko00760	Nicotinate and nicotinamide metabolism	7
ko00670	One carbon pool by folate	2
ko00770	Pantothenate and CoA biosynthesis	3
ko00860	Porphyrin and chlorophyll metabolism	2
ko00740	Riboflavin metabolism	1
ko00130	Ubiquinone and other terpenoid-quinone biosynthesis	1
ko00460	Cyanoamino acid metabolism	2
ko00480	Glutathione metabolism	5
ko00450	Selenocompound metabolism	1
ko00430	Taurine and hypotaurine metabolism	2
ko00410	beta-Alanine metabolism	1
ko00909	Sesquiterpenoid and triterpenoid biosynthesis	1
ko00230	Purine metabolism	10
ko00240	Pyrimidine metabolism	12

Table S10. Prediction of the transcription factors in the blue module;

Type	Family	ID	KME
PK	AGC_NDR	gene_1544	0.965052721
PK	AGC_PDK1	gene_8134	-0.879752768
PK	AGC_PKA-PKG	gene_5067	0.97382954
PK	AGC_RSK-2	gene_3756	0.865341264
PK	AGC-Pl	gene_9051	0.928335061
TF	bHLH	gene_5211	0.883890505
TF	C2H2	gene_9414	0.96833764
TF	C2H2	gene_1821	0.942326713
TF	C2H2	gene_3145	0.930651509
TF	C2H2	gene_6545	0.92328757
TF	C2H2	gene_1224	0.871598715
TF	C2H2	gene_9406	-0.895580891
TF	C3H	gene_379	0.96419166
TF	C3H	gene_10549	0.944729247
TF	C3H	gene_10879	-0.94758573
PK	CAMK_AMPK	gene_4006	0.973466648
PK	CAMK_AMPK	gene_9848	0.909358777
PK	CAMK_CAMKL-CHK1	gene_6267	-0.939171035
PK	CAMK_CDPK	gene_5955	0.876311694
PK	CAMK_CDPK	gene_10825	0.862846156
PK	CAMK_CDPK	gene_1289	-0.887141995
PK	CAMK_OST1L	gene_2419	0.905962921
PK	CAMK_OST1L	gene_5765	0.880109108
PK	CK1_CK1	gene_2096	0.922067812
PK	CMGC_CDK-CDK8	gene_12029	0.887421555
PK	CMGC_CDK-PITSLRE	gene_7462	0.892928932
PK	CMGC_CK2	gene_10591	0.869603534
PK	CMGC_DYRK-PRP4	gene_7689	0.897342714
PK	CMGC_MAPK	gene_8668	0.926357452
TR	GNAT	gene_7921	0.946715236
TR	GNAT	gene_8780	0.937123557
TR	GNAT	gene_2866	0.897106952
TF	HB-other	gene_2391	-0.902130564
TR	HMG	gene_2844	0.836803669
TF	HSF	gene_10661	-0.914930041
TR	IWS1	gene_9806	0.921520471
TR	IWS1	gene_314	0.825080221
TR	Jumonji	gene_8942	0.924545339
TF	LIM	gene_10949	0.881332561
TF	MADS-M-type	gene_10611	0.861362825
TR	MED6	gene_8446	0.913140835
TR	MED6	gene_4600	0.909866293
TF	NF-X1	gene_5458	-0.933123125
TR	Others	gene_7407	0.931764702
TR	Others	gene_2628	0.904712496
PK	PEK_GCN2	gene_4145	0.926410074
TR	PHD	gene_1737	0.964575012
TR	PHD	gene_40	0.954434918
TR	Rcd1-like	gene_11200	0.918270726
TR	SET	gene_8246	0.957991141
TR	SET	gene_2826	0.932171665
TR	SET	gene_8224	0.907668868
TR	SNF2	gene_11087	0.968214388
TR	SNF2	gene_7234	0.954244352
TR	SNF2	gene_3897	0.938166754
TR	SNF2	gene_7041	0.925765176
TR	SNF2	gene_827	0.89868409

TR	SNF2	gene_4074	0.866004334
TR	SNF2	gene_2122	0.861850815
PK	STE_STE11	gene_2203	0.817323361
PK	STE_STE20-Pl	gene_2817	0.948575494
PK	STE_STE20-YSK	gene_843	0.922171899
PK	STE_STE-Pl	gene_7562	0.83288494
TR	SWI/SNF-BAF60b	gene_4697	0.895394247
PK	TKL-Cr-4	gene_8053	0.843659217
PK	TKL-Pl-4	gene_7871	0.843587549
TR	TRAF	gene_10898	0.943211745
TR	TRAF	gene_1201	-0.929538937
TF	zn-clus	gene_3383	0.959768787
TF	zn-clus	gene_6727	0.952794627
TF	zn-clus	gene_3192	0.896426228
TF	zn-clus	gene_8774	0.822403249
TF	zn-clus	gene_11776	-0.853851199
TF	zn-clus	gene_1322	-0.886491806

Table S11. GO term enrichment of the hub genes in the turquoise module

Catogry	GO.ID	Term	Annotated	Significant	Expected	KS
BP	GO:0006605	protein targeting	85	2	1.57	0.0051
BP	GO:0072594	establishment of protein localization to organelle	86	2	1.59	0.0069
BP	GO:0051603	proteolysis involved in cellular protein catabolic process	78	1	1.44	0.0099
BP	GO:0019941	modification-dependent protein catabolic process	73	1	1.35	0.0106
BP	GO:0051646	mitochondrion localization	14	1	0.26	0.0125
BP	GO:0006511	ubiquitin-dependent protein catabolic process	72	1	1.33	0.0142
BP	GO:0006612	protein targeting to membrane	20	1	0.37	0.0166
BP	GO:0040020	regulation of meiosis	8	2	0.15	0.0188
BP	GO:0043632	modification-dependent macromolecule catabolic process	85	1	1.57	0.0193
BP	GO:0071840	cellular component organization or biogenesis	679	22	12.52	0.0264
BP	GO:0034622	cellular macromolecular complex assembly	159	5	2.93	0.0279
BP	GO:0065003	macromolecular complex assembly	178	5	3.28	0.0316
BP	GO:0007005	mitochondrion organization	82	2	1.51	0.0334
BP	GO:0006623	protein targeting to vacuole	25	1	0.46	0.037
BP	GO:0072666	establishment of protein localization to vacuole	25	1	0.46	0.037
BP	GO:0006996	organelle organization	409	17	7.54	0.0404
BP	GO:1902580	single-organism cellular localization	59	3	1.09	0.0413
BP	GO:1902578	single-organism localization	59	3	1.09	0.0413
BP	GO:0016043	cellular component organization	571	22	10.53	0.043
BP	GO:0060631	regulation of meiosis I	4	2	0.07	0.0447
BP	GO:0017004	cytochrome complex assembly	8	1	0.15	0.0482
CC	GO:0005741	mitochondrial outer membrane	28	1	0.53	0.002
CC	GO:0070461	SAGA-type complex	14	2	0.27	0.0026
CC	GO:0044430	cytoskeletal part	122	7	2.33	0.0106
CC	GO:0071014	post-mRNA release spliceosomal complex	3	1	0.06	0.0251
CC	GO:0005856	cytoskeleton	127	7	2.42	0.0286
CC	GO:0000124	SAGA complex	9	1	0.17	0.0369
CC	GO:0005768	endosome	38	1	0.72	0.0397
CC	GO:0000813	ESCRT I complex	3	1	0.06	0.0419
CC	GO:0000775	chromosome, centromeric region	38	6	0.72	0.0429
CC	GO:0015629	actin cytoskeleton	33	2	0.63	0.0452
CC	GO:0010008	endosome membrane	15	1	0.29	0.0453
CC	GO:0034506	chromosome, centromeric core domain	6	1	0.11	0.0457
CC	GO:0005622	intracellular	2162	50	41.24	0.0482

CC	GO:0000777	condensed chromosome kinetochore	16	5	0.31	0.0484
MF	GO:0003723	RNA binding	246	4	3.86	0.003
MF	GO:0000104	succinate dehydrogenase activity	4	3	0.06	0.0125
MF	GO:0016620	oxidoreductase activity, acting on the aldehyde or oxo group of donors,	20	1	0.31	0.0135
MF	GO:0016635	oxidoreductase activity, acting on the CH-CH group of donors, quinone or [illegible]	3	2	0.05	0.017
MF	GO:0008177	succinate dehydrogenase (ubiquinone) activity	2	2	0.03	0.0189
MF	GO:0004003	ATP-dependent DNA helicase activity	12	2	0.19	0.0208
MF	GO:0004180	carboxypeptidase activity	25	1	0.39	0.021
MF	GO:0008238	exopeptidase activity	47	1	0.74	0.0254
MF	GO:0016903	oxidoreductase activity, acting on the aldehyde or oxo group of donors	27	1	0.42	0.0339
MF	GO:0050660	flavin adenine dinucleotide binding	62	2	0.97	0.0341
MF	GO:0008135	translation factor activity, nucleic acid binding	60	1	0.94	0.0391
MF	GO:0070008	serine-type exopeptidase activity	20	1	0.31	0.0498

Table S12. KEGG pathway enrichment of the hub genes in the turquoise module

Kegg_pathway	ko_id	P-value	rich_factor
Cell cycle - yeast	ko04111	5.10538E-05	5.618342152
Citrate cycle (TCA cycle)	ko00020	0.001054943	8.427513228
Meiosis - yeast	ko04113	0.015370066	4.063265306
Oxidative phosphorylation	ko00190	0.047259252	2.880289331
Nicotinate and nicotinamide metabolism	ko00760	0.05566722	5.171428571
Basal transcription factors	ko03022	0.101649316	3.670046083
Carbon metabolism	ko01200	0.106282893	2.187912088
Selenocompound metabolism	ko00450	0.116918093	8.126530612
Regulation of mitophagy - yeast	ko04139	0.118741827	3.346218487
Ribosome biogenesis in eukaryotes	ko03008	0.134396878	2.33776908
Non-homologous end-joining	ko03450	0.177647422	5.171428571
Sulfur relay system	ko04122	0.177647422	5.171428571
Biosynthesis of antibiotics	ko01130	0.182356654	1.551428571
Spliceosome	ko03040	0.246057966	1.741399417
Arginine biosynthesis	ko00220	0.247873995	3.555357143
Sulfur metabolism	ko00920	0.26120281	3.346218487
Porphyrin and chlorophyll metabolism	ko00860	0.274302051	3.16031746
Ubiquitin mediated proteolysis	ko04120	0.284727337	1.896190476
Fructose and mannose metabolism	ko00051	0.312260416	2.708843537
Pentose and glucuronate interconversions	ko00040	0.359883472	2.275428571
Pyrimidine metabolism	ko00240	0.382729549	1.516952381
RNA polymerase	ko03020	0.435627177	1.777678571
Arginine and proline metabolism	ko00330	0.465354545	1.625306122
Cysteine and methionine metabolism	ko00270	0.520314965	1.387456446
Nucleotide excision repair	ko03420	0.520314965	1.387456446
Biosynthesis of amino acids	ko01230	0.585416763	1.034285714
Starch and sucrose metabolism	ko00500	0.628069018	1.034285714
mRNA surveillance pathway	ko03015	0.666680387	0.932552693
Endocytosis	ko04144	0.732571108	0.77925636
Protein processing in endoplasmic reticulum	ko04141	0.764965422	0.711071429
Purine metabolism	ko00230	0.80471819	0.632063492
RNA transport	ko03013	0.857807242	0.53164219

Table S13. KEGG pathway classification of the hub genes in the turquoise module

KEGG	ko.ID	KEGG Category2
Cellular Processes(9）	ko04111	Cell cycle - yeast（8）
	ko04113	Meiosis - yeast（4）
	ko04144	Endocytosis（1）
Genetic（15） Information Processing	ko04141	Protein processing in endoplasmic reticulum（1）
	ko04122	Sulfur relay system（1）
	ko04120	Ubiquitin mediated proteolysis（2）
	ko03450	Non-homologous end-joining（1）
	ko03420	Nucleotide excision repair（1）
	ko03022	Basal transcription factors（2）
	ko03020	RNA polymerase（1）
	ko03040	Spliceosome（3）
	ko03013	RNA transport（1）
	ko03008	Ribosome biogenesis in eukaryotes（3）
	ko03015	mRNA surveillance pathway（1）
Metabolism（13）	ko00330	Arginine and proline metabolism(1)
	ko00220	Arginine biosynthesis(1)
	ko00270	Cysteine and methionine metabolism(1)
	ko00020	Citrate cycle (TCA cycle)(4)
	ko00051	Fructose and mannose metabolism(1)
	ko00040	Pentose and glucuronate interconversions(1)
	ko00500	Starch and sucrose metabolism(1)
	ko00190	Oxidative phosphorylation(4)
	ko00920	Sulfur metabolism(1)
	ko01230	Biosynthesis of amino acids(2)
	ko01130	Biosynthesis of antibiotics(6)
	ko01200	Carbon metabolism(4)
	ko00760	Nicotinate and nicotinamide metabolism(2)
	ko00860	Porphyrin and chlorophyll metabolism(1)
	ko00450	Selenocompound metabolism(1)
	ko00230	Purine metabolism(1)
	ko00240	Pyrimidine metabolism(2)

Table S14. Prediction of the transcription factors in the turquoise module

Type	Family	ID	gene_name	KME
PK	CMGC_CK2	gene_10591	jgi.p|Tragib1|913171	-0.81255124
TR	GNAT	gene_7921	jgi.p|Tragib1|1420749	-0.856262211
PK	PEK_GCN2	gene_4145	jgi.p|Tragib1|1316322	-0.811506802
TR	PHD	gene_40	jgi.p|Tragib1|1308157	-0.846670246
TR	SNF2	gene_11087	jgi.p|Tragib1|1400843	-0.871475649
TR	TRAF	gene_10898	jgi.p|Tragib1|1399858	-0.891335236
TF	zn-clus	gene_6727	jgi.p|Tragib1|1419700	-0.867827313

Table S15. Gene expression changes of secondary metabolites in DEGs at different time period.

**ID**	**CKvsMX1 log2FC**	**CKvsMX2 log2FC**	**CKvsMX3 log2FC**	**CKvsMX4 log2FC**	**KOG_class_annotation**	**Swiss_Prot_annotation**
gene_356	1.14	1.24	1.18	1.33	Inorganic ion transport and metabolism;; Secondary metabolites biosynthesis, transport and catabolism	Ferric reductase transmembrane component 3 OS=Saccharomyces cerevisiae (strain ATCC 204508 / S288c) OX=559292 GN=FRE3 PE=1 SV=1
gene_3902	1.47	1.92	1.95	1.96	Secondary metabolites biosynthesis, transport and catabolism	Laccase-1 OS=Trametes villosa OX=47662 GN=LCC1 PE=3 SV=1
gene_9080	1.56	1.46	1.32	1.01	Secondary metabolites biosynthesis, transport and catabolism;; General function prediction only	2-oxoglutarate-Fe(II) type oxidoreductase OS=Neosartorya fumigata (strain ATCC MYA-4609 / Af293 / CBS 101355 / FGSC A1100) OX=330879 GN=encD PE=1 SV=2
gene_11416	2.69	2.49	2.75	3.01	Secondary metabolites biosynthesis, transport and catabolism	Peroxisomal hydratase-dehydrogenase-epimerase OS=Saccharomyces cerevisiae (strain ATCC 204508 / S288c) OX=559292 GN=FOX2 PE=1 SV=1
gene_3807	1.05	#N/A	#N/A	#N/A	Secondary metabolites biosynthesis, transport and catabolism;; General function prediction only	Oxidoreductase vrtI OS=Penicillium aethiopicum OX=36650 GN=vrtI PE=1 SV=1
gene_3906	1.25	1.26	#N/A	1.27	Secondary metabolites biosynthesis, transport and catabolism	NADPH-dependent 1-acyldihydroxyacetone phosphate reductase OS=Schizosaccharomyces pombe (strain 972 / ATCC 24843) OX=284812 GN=ayr1 PE=3 SV=2
gene_10950	1.60	1.44	1.32	#N/A	Secondary metabolites biosynthesis, transport and catabolism;; Lipid transport and metabolism	Cytochrome P450 monooxygenase FUM15 OS=Gibberella moniliformis (strain M3125 / FGSC 7600) OX=334819 GN=FUM15 PE=3 SV=1

gene_9797	#N/A	1.41	1.35	1.32	Secondary metabolites biosynthesis, transport and catabolism	Dual-functional monooxygenase/methyltransferase psoF OS=Neosartorya fumigata (strain ATCC MYA-4609 / Af293 / CBS 101355 / FGSC A1100) OX=330879 GN=psoF PE=1 SV=1
gene_9841	#N/A	1.61	1.81	2.18	Secondary metabolites biosynthesis, transport and catabolism	Uncharacterized oxidoreductase C162.03 OS=Schizosaccharomyces pombe (strain 972 / ATCC 24843) OX=284812 GN=SPCC162.03 PE=3 SV=1
gene_2042	#N/A	#N/A	1.53	1.77	Secondary metabolites biosynthesis, transport and catabolism	--
gene_6377	#N/A	#N/A	3.20	#N/A	Inorganic ion transport and metabolism;; Secondary metabolites biosynthesis, transport and catabolism	Ferric reductase transmembrane component 1 OS=Schizosaccharomyces pombe (strain 972 / ATCC 24843) OX=284812 GN=frp1 PE=1 SV=1
gene_8611	#N/A	#N/A	4.49	#N/A	--	Manganese peroxidase 3 OS=Phlebia radiata OX=5308 GN=mnp3 PE=2 SV=1
gene_4408	#N/A	#N/A	1.27	#N/A	Secondary metabolites biosynthesis, transport and catabolism;; General function prediction only	Oxidoreductase vrtI OS=Penicillium aethiopicum OX=36650 GN=vrtI PE=1 SV=1
gene_10265	#N/A	#N/A	#N/A	1.46	Secondary metabolites biosynthesis, transport and catabolism	Iron-sulfur clusters transporter atm1, mitochondrial OS=Aspergillus oryzae (strain ATCC 42149 / RIB 40) OX=510516 GN=atm1 PE=3 SV=1
gene_4543	#N/A	#N/A	#N/A	3.19	Secondary metabolites biosynthesis, transport and catabolism	Multifunctional cytochrome P450 monooxygenase af510 OS=Neosartorya fumigata (strain ATCC MYA-4609 / Af293 / CBS 101355 / FGSC A1100) OX=330879 GN=af510 PE=1 SV=1
gene_5152	#N/A	#N/A	#N/A	1.00	Secondary metabolites biosynthesis, transport and catabolism	Cytochrome P450 monooxygenase psiH OS=Psilocybe cubensis OX=181762 GN=psiH PE=1 SV=1
gene_7	#N/A	#N/A	#N/A	1.20	Secondary metabolites biosynthesis, transport and catabolism	Cytochrome P450 monooxygenase psiH OS=Psilocybe cubensis OX=181762 GN=psiH PE=1 SV=1
gene_2828	#N/A	#N/A	#N/A	1.10	Secondary metabolites biosynthesis, transport and catabolism	Corticosteroid-binding protein OS=Candida albicans (strain SC5314 / ATCC MYA-2876) OX=237561 GN=CBP1 PE=1 SV=2
gene_9018	#N/A	#N/A	#N/A	1.17	Carbohydrate transport and metabolism;; Secondary metabolites biosynthesis, transport and catabolism	D-xylose 1-dehydrogenase (NADP(+)) OS=Hypocrea jecorina (strain ATCC 56765 / BCRC 32924 / NRRL 11460 / Rut C-30) OX=1344414 GN=xyd1 PE=1 SV=1
gene_768	#N/A	#N/A	#N/A	1.06	Secondary metabolites biosynthesis, transport and catabolism	3-ketodihydrosphingosine reductase TSC10 OS=Cryptococcus neoformans var. neoformans serotype D (strain B-3501A) OX=283643 GN=TSC10 PE=3 SV=1
gene_5434	-2.11	-1.82	-1.59	-1.57	Secondary metabolites biosynthesis, transport and catabolism	--
gene_6187	-1.56	#N/A	-1.71	-2.20	Secondary metabolites biosynthesis, transport and catabolism;; General function prediction only	Oxidoreductase vrtI OS=Penicillium aethiopicum OX=36650 GN=vrtI PE=1 SV=1
gene_11232	-1.29	-1.16	-1.15	-1.02	Secondary metabolites biosynthesis, transport and catabolism	Metal resistance protein YCF1 OS=Saccharomyces cerevisiae (strain ATCC 204508 / S288c) OX=559292 GN=YCF1 PE=1 SV=2
gene_8967	-1.50	-2.09	-2.07	-2.28	Secondary metabolites biosynthesis, transport and catabolism	Oligomycin resistance ATP-dependent permease YOR1 OS=Saccharomyces cerevisiae (strain ATCC 204508 / S288c) OX=559292 GN=YOR1 PE=1 SV=1

gene_420	-1.02	#N/A	#N/A	#N/A	Secondary metabolites biosynthesis, transport and catabolism	ATP-dependent bile acid permease OS=Saccharomyces cerevisiae (strain ATCC 204508 / S288c) OX=559292 GN=YBT1 PE=1 SV=2
gene_8398	-1.42	-1.76	-1.74	-1.78	Secondary metabolites biosynthesis, transport and catabolism	Leptomycin B resistance protein pmd1 OS=Schizosaccharomyces pombe (strain 972 / ATCC 24843) OX=284812 GN=pmd1 PE=3 SV=2
gene_5257	-2.40	-2.51	-2.50	-3.12	Secondary metabolites biosynthesis, transport and catabolism	Brefeldin A resistance protein OS=Schizosaccharomyces pombe (strain 972 / ATCC 24843) OX=284812 GN=bfr1 PE=1 SV=1
gene_3875	-5.10	-2.20	-2.86	#N/A	Secondary metabolites biosynthesis, transport and catabolism	NADP-dependent alcohol dehydrogenase 7 OS=Saccharomyces cerevisiae (strain ATCC 204508 / S288c) OX=559292 GN=ADH7 PE=1 SV=1
gene_9728	-1.47	-1.20	#N/A	#N/A	Secondary metabolites biosynthesis, transport and catabolism	Probable D-xylulose reductase A OS=Neosartorya fumigata (strain ATCC MYA-4609 / Af293 / CBS 101355 / FGSC A1100) OX=330879 GN=xdhA PE=3 SV=2
gene_757	#N/A	#N/A	-1.47	-1.19	Secondary metabolites biosynthesis, transport and catabolism	D-xylulose reductase A OS=Aspergillus niger OX=5061 GN=xdhA PE=3 SV=1
gene_7840	-1.85	-1.98	-2.25	-2.24	Secondary metabolites biosynthesis, transport and catabolism	Alcohol dehydrogenase 1 OS=Kluyveromyces marxianus OX=4911 GN=ADH1 PE=3 SV=1
gene_11568	#N/A	#N/A	-2.11	-2.01	Secondary metabolites biosynthesis, transport and catabolism	NADP-dependent alcohol dehydrogenase 6 OS=Saccharomyces cerevisiae (strain ATCC 204508 / S288c) OX=559292 GN=ADH6 PE=1 SV=1
gene_7760	#N/A	#N/A	-1.39	-1.36	Secondary metabolites biosynthesis, transport and catabolism	NADP-dependent alcohol dehydrogenase 6 OS=Saccharomyces cerevisiae (strain ATCC 204508 / S288c) OX=559292 GN=ADH6 PE=1 SV=1
gene_9319	-1.10	#N/A	#N/A	#N/A	Secondary metabolites biosynthesis, transport and catabolism	Adenylate-forming reductase 06235 OS=Coprinopsis cinerea (strain Okayama-7 / 130 / ATCC MYA-4618 / FGSC 9003) OX=240176 GN=CC1G_06235 PE=2 SV=3
gene_9779	-3.54	-3.16	-3.67	-3.43	Secondary metabolites biosynthesis, transport and catabolism	Adenylate-forming reductase NpS11 OS=Heterobasidion annosum OX=13563 GN=npS11 PE=2 SV=1
gene_2142	-1.13	#N/A	#N/A	#N/A	Secondary metabolites biosynthesis, transport and catabolism	Nonribosomal peptide synthetase 2 OS=Neosartorya fumigata (strain ATCC MYA-4609 / Af293 / CBS 101355 / FGSC A1100) OX=330879 GN=NRPS2 PE=2 SV=1
gene_7203	#N/A	#N/A	-1.02	#N/A	Secondary metabolites biosynthesis, transport and catabolism	Peroxisomal primary amine oxidase OS=Pichia angusta OX=870730 GN=AMO PE=1 SV=1
gene_11123	-1.39	-1.59	-1.75	#N/A	Secondary metabolites biosynthesis, transport and catabolism;; Lipid transport and metabolism	Cytochrome P450 monooxygenase FUM15 OS=Gibberella moniliformis (strain M3125 / FGSC 7600) OX=334819 GN=FUM15 PE=3 SV=1
gene_11279	-1.88	-1.58	-1.53	-1.37	Secondary metabolites biosynthesis, transport and catabolism	Cytochrome P450 monooxygenase psiH OS=Psilocybe cubensis OX=181762 GN=psiH PE=1 SV=1
gene_3855	-1.21	-1.35	-1.38	-1.04	Secondary metabolites biosynthesis, transport and catabolism	Ent-kaurene oxidase OS=Gibberella fujikuroi OX=5127 GN=CYP503A1 PE=1 SV=1
gene_3857	-1.07	-1.05	#N/A	#N/A	Secondary metabolites biosynthesis, transport and catabolism;; Lipid transport and metabolism	Ent-kaurene oxidase OS=Gibberella intermedia OX=948311 GN=CYP503A1 PE=1 SV=1
gene_4049	-1.24	-1.48	-1.48	-1.03	Secondary metabolites biosynthesis, transport and catabolism;; Lipid transport and metabolism	Cytochrome P450 monooxygenase FUM15 OS=Gibberella moniliformis (strain M3125 / FGSC 7600) OX=334819 GN=FUM15 PE=3 SV=1

gene_4296	-1.32	-1.39	-1.90	#N/A	Secondary metabolites biosynthesis, transport and catabolism;; Lipid transport and metabolism	Cytochrome P450 monooxygenase FUM15 OS=Gibberella moniliformis (strain M3125 / FGSC 7600) OX=334819 GN=FUM15 PE=3 SV=1
gene_4378	#N/A	-1.13	-1.23	-1.14	Secondary metabolites biosynthesis, transport and catabolism;; Lipid transport and metabolism	Cytochrome P450 monooxygenase FUM15 OS=Gibberella moniliformis (strain M3125 / FGSC 7600) OX=334819 GN=FUM15 PE=3 SV=1
gene_4487	-4.06	-6.00	-5.36	-3.40	Secondary metabolites biosynthesis, transport and catabolism;; Lipid transport and metabolism	Cytochrome P450 monooxygenase FUM15 OS=Gibberella moniliformis (strain M3125 / FGSC 7600) OX=334819 GN=FUM15 PE=3 SV=1
gene_6093	-1.52	-1.85	-2.12	-1.65	Secondary metabolites biosynthesis, transport and catabolism	Cytochrome P450 monooxygenase psiH OS=Psilocybe cubensis OX=181762 GN=psiH PE=1 SV=1
gene_6115	-1.79	-2.34	-1.96	-1.81	Secondary metabolites biosynthesis, transport and catabolism;; Lipid transport and metabolism	Cytochrome P450 monooxygenase FUM15 OS=Gibberella moniliformis (strain M3125 / FGSC 7600) OX=334819 GN=FUM15 PE=3 SV=1
gene_6568	-1.17	-1.30	-1.56	-1.50	Secondary metabolites biosynthesis, transport and catabolism	Benzoate 4-monooxygenase OS=Aspergillus niger OX=5061 GN=bphA PE=1 SV=1
gene_6883	-1.09	-1.00	-1.27	-1.24	Secondary metabolites biosynthesis, transport and catabolism;; Lipid transport and metabolism	Cytochrome P450 52A10 OS=Candida maltosa OX=5479 GN=CYP52A10 PE=2 SV=1
gene_7048	#N/A	#N/A	-1.07	-1.03	Secondary metabolites biosynthesis, transport and catabolism	Cytochrome P450 monooxygenase psiH OS=Psilocybe cubensis OX=181762 GN=psiH PE=1 SV=1
gene_7521	-2.44	-2.49	-2.65	-1.19	Secondary metabolites biosynthesis, transport and catabolism;; Lipid transport and metabolism	Bifunctional cytochrome P450/NADPH--P450 reductase OS=Aspergillus oryzae (strain ATCC 42149 / RIB 40) OX=510516 GN=CYP505A3 PE=2 SV=1
gene_7628	-2.01	-1.78	-1.79	-1.65	Secondary metabolites biosynthesis, transport and catabolism;; Lipid transport and metabolism	Cytochrome P450 monooxygenase FUM15 OS=Gibberella moniliformis (strain M3125 / FGSC 7600) OX=334819 GN=FUM15 PE=3 SV=1
gene_7660	-1.05	#N/A	-1.06	-1.19	Secondary metabolites biosynthesis, transport and catabolism	Cytochrome P450 monooxygenase FUM15 OS=Gibberella moniliformis (strain M3125 / FGSC 7600) OX=334819 GN=FUM15 PE=3 SV=1
gene_8493	-1.49	-1.58	-1.24	-1.46	Secondary metabolites biosynthesis, transport and catabolism	Cytochrome P450 monooxygenase FUM15 OS=Gibberella moniliformis (strain M3125 / FGSC 7600) OX=334819 GN=FUM15 PE=3 SV=1
gene_8659	#N/A	-2.08	-1.64	#N/A	Secondary metabolites biosynthesis, transport and catabolism;; Lipid transport and metabolism	Cytochrome P450 monooxygenase FUM15 OS=Gibberella moniliformis (strain M3125 / FGSC 7600) OX=334819 GN=FUM15 PE=3 SV=1
gene_8661	-1.16	#N/A	#N/A	#N/A	Secondary metabolites biosynthesis, transport and catabolism;; Lipid transport and metabolism	Cytochrome P450 monooxygenase FUM15 OS=Gibberella moniliformis (strain M3125 / FGSC 7600) OX=334819 GN=FUM15 PE=3 SV=1
gene_9113	-4.21	-3.30	-3.47	-4.09	Secondary metabolites biosynthesis, transport and catabolism;; Lipid transport and metabolism	Cytochrome P450 monooxygenase FUM15 OS=Gibberella moniliformis (strain M3125 / FGSC 7600) OX=334819 GN=FUM15 PE=3 SV=1
gene_9172	#N/A	#N/A	#N/A	-1.48	Secondary metabolites biosynthesis, transport and catabolism	Cytochrome P450 monooxygenase patI OS=Aspergillus clavatus (strain ATCC 1007 / CBS 513.65 / DSM 816 / NCTC 3887 / NRRL 1) OX=344612 GN=patI PE=1 SV=2
gene_9592	#N/A	-1.37	#N/A	#N/A	Secondary metabolites biosynthesis, transport and catabolism	Cytochrome P450 monooxygenase psiH OS=Psilocybe cubensis OX=181762 GN=psiH PE=1 SV=1

gene_9718	-2.01	#N/A	#N/A	#N/A	Secondary metabolites biosynthesis, transport and catabolism	Cytochrome P450 monooxygenase FCK2 OS=Fusarium pseudograminearum (strain CS3096) OX=1028729 GN=FCK2 PE=2 SV=1
gene_559	-5.33	-4.92	-2.59	-4.02	--	Dye-decolorizing peroxidase msp1 OS=Mycetinis scorodonius OX=182058 GN=msp1 PE=1 SV=1
gene_561	-3.22	-2.97	-2.19	-2.36	--	Peroxidase 2 OS=Mycetinis scorodonius OX=182058 GN=msp2 PE=1 SV=1
gene_562	#N/A	#N/A	-1.49	#N/A	--	Peroxidase 2 OS=Mycetinis scorodonius OX=182058 GN=msp2 PE=1 SV=1
gene_2184	-2.36	-1.75	-2.06	-2.07	Secondary metabolites biosynthesis, transport and catabolism	Glutathione synthetase OS=Saccharomyces cerevisiae (strain ATCC 204508 / S288c) OX=559292 GN=GSH2 PE=1 SV=1
gene_4163	#N/A	#N/A	#N/A	-1.91	Inorganic ion transport and metabolism;; Secondary metabolites biosynthesis, transport and catabolism	Probable ferric reductase transmembrane component OS=Candida albicans OX=5476 GN=CFL1 PE=3 SV=1
gene_11537	#N/A	-1.15	-1.24	#N/A	--	Versatile peroxidase VPL2 OS=Pleurotus eryngii OX=5323 GN=vpl2 PE=1 SV=1
gene_713	#N/A	-2.05	-2.48	-2.82	--	Versatile peroxidase VPL1 OS=Pleurotus eryngii OX=5323 GN=vpl1 PE=1 SV=1
gene_2141	-1.68	-1.63	-1.86	-1.90	Secondary metabolites biosynthesis, transport and catabolism	L-ornithine N(5)-monooxygenase OS=Neosartorya fumigata (strain ATCC MYA-4609 / Af293 / CBS 101355 / FGSC A1100) OX=330879 GN=sidA PE=1 SV=1
gene_1741	-1.04	#N/A	-1.17	-1.24	Secondary metabolites biosynthesis, transport and catabolism	Laccase-3 OS=Trametes villosa OX=47662 GN=LCC3 PE=3 SV=1
gene_11877	-3.57	-2.82	-3.21	-1.52	Secondary metabolites biosynthesis, transport and catabolism;; General function prediction only	2-oxoglutarate-Fe(II) type oxidoreductase hxnY OS=Emericella nidulans (strain FGSC A4 / ATCC 38163 / CBS 112.46 / NRRL 194 / M139) OX=227321 GN=hxnY PE=2 SV=1
gene_4407	-1.63	-1.47	-1.64	-1.81	Secondary metabolites biosynthesis, transport and catabolism;; General function prediction only	--
gene_9190	-1.35	-1.38	-1.57	-1.99	Secondary metabolites biosynthesis, transport and catabolism;; General function prediction only	--
gene_3379	#N/A	#N/A	-1.03	#N/A	--	Cytochrome c peroxidase, mitochondrial OS=Cryptococcus neoformans var. neoformans serotype D (strain JEC21 / ATCC MYA-565) OX=214684 GN=CCP1 PE=3 SV=1
gene_2574	#N/A	#N/A	-1.32	#N/A	Energy production and conversion;; Lipid transport and metabolism;; Secondary metabolites biosynthesis, transport and catabolism	Acyl carrier protein, mitochondrial OS=Neurospora crassa (strain ATCC 24698 / 74-OR23-1A / CBS 708.71 / DSM 1257 / FGSC 987) OX=367110 GN=nuo-12 PE=1 SV=2
gene_10018	-3.16	-2.67	-3.52	-2.82	Secondary metabolites biosynthesis, transport and catabolism	Uncharacterized oxidoreductase C162.03 OS=Schizosaccharomyces pombe (strain 972 / ATCC 24843) OX=284812 GN=SPCC162.03 PE=3 SV=1
gene_990	-1.36	-1.53	-1.70	-1.27	Secondary metabolites biosynthesis, transport and catabolism	Dehydrogenase RED2 OS=Cochliobolus heterostrophus (strain C4 / ATCC 48331 / race T) OX=665024 GN=RED2 PE=3 SV=1

gene_10005	-2.49	-2.37	-3.29	-2.91	Secondary metabolites biosynthesis, transport and catabolism	Uncharacterized oxidoreductase C162.03 OS=Schizosaccharomyces pombe (strain 972 / ATCC 24843) OX=284812 GN=SPCC162.03 PE=3 SV=1
gene_1269	#N/A	-1.13	-1.30	#N/A	Secondary metabolites biosynthesis, transport and catabolism	Uncharacterized oxidoreductase C162.03 OS=Schizosaccharomyces pombe (strain 972 / ATCC 24843) OX=284812 GN=SPCC162.03 PE=3 SV=1
gene_11466	-3.04	-3.08	-3.59	-3.39	Secondary metabolites biosynthesis, transport and catabolism	Alcohol dehydrogenase 1 OS=Scheffersomyces stipitis (strain ATCC 58785 / CBS 6054 / NBRC 10063 / NRRL Y-11545) OX=322104 GN=ADH1 PE=3 SV=1
